# BDNF-loaded PDADMAC-heparin multilayers: a novel approach for neuroblastoma cell study

**DOI:** 10.1038/s41598-023-45045-y

**Published:** 2023-10-20

**Authors:** Maria Dąbkowska, Iga Stukan, Bogusław Kowalski, Wiktoria Donerowicz, Monika Wasilewska, Alicja Szatanik, Małgorzata Stańczyk-Dunaj, Aneta Michna

**Affiliations:** 1 Independent Laboratory of Pharmacokinetic and Clinical Pharmacy, Pomeranian Medical University, Rybacka 1, 70-204 Szczecin, Poland; 2 Department of General Pathology, Pomeranian Medical University, Rybacka 1, 70-204 Szczecin, Poland; 3 Jerzy Haber Institute of Catalysis and Surface Chemistry, Polish Academy of Sciences, Niezapominajek 8, 30-239 Kraków, Poland; 4 Department of Medical Chemistry, Pomeranian Medical University, Rybacka 1, 70-204 Szczecin, Poland

**Keywords:** Biotechnology, Chemical biology, Drug discovery, Chemistry, Materials science, Nanoscience and technology, Cancer

## Abstract

Biomaterial science has contributed tremendously to developing nanoscale materials for delivering biologically active compounds, enhancing protein stability, and enabling its therapeutic use. This paper presents a process of formation of polyelectrolyte multilayer (PEM) prepared by sequential adsorption of positively charged polydiallyldimethylammonium chloride (PDADMAC) and negatively charged heparin sodium salt (HP), from low polyelectrolyte concentration, on a solid substrate. PEM was further applied as a platform for the adsorption of a brain-derived growth factor (BDNF), which is a protein capable of regulating neuronal cell development. The multilayers containing BDNF were thoroughly characterized by electrokinetic (streaming potential measurements, SPM) and optical (optical waveguide lightmode spectroscopy, OWLS) techniques. It was found that BDNF was significantly adsorbed onto polyelectrolyte multilayers terminated by HP under physiological conditions. We further explore the effect of established PEMs in vitro on the neuroblastoma SH-SY5Y cell line. An enzyme-linked immunosorbent assay (ELISA) confirmed that BDNF was released from multilayers, and the use of the PEMs intensified its cellular uptake. Compared to the control, PEMs with adsorbed BDNF significantly reduced cell viability and mitochondrial membrane polarization to as low as 72% and 58%, respectively. HPLC analysis showed that both PDADMAC-terminated and HP-terminated multilayers have antioxidative properties as they almost by half decreased lipid peroxidation in SH-SY5Y cells. Finally, enhanced formation of spheroid-like, 3D structures was observed by light microscopy. We offer a well-characterized PEM with antioxidant properties acting as a BDNF carrier, stabilizing BDNF and making it more accessible to cells in an inhomogeneous, dynamic, and transient in vitro environment. Described multilayers can be utilized in future biomedical applications, such as boosting the effect of treatment by selective anticancer as adjuvant therapy, and in biomedical research for future development of more precise neurodegenerative disease models, as they enhance cellular 3D structure formation.

## Introduction

In recent decades, nanobiotechnology has contributed tremendously to advancing and developing nanoscale materials for drug-delivery cancer therapies to enhance the specificity of drug actions and reduce systemic side effects. Significant progress has been made in understanding the molecular causes of neuronal cell damage has been documented in recent decades, leading to the development of innovative methods for treating and preventing neurodegenerative illnesses^1–3^, including such as aggressive malignancy such as neuroblastoma.

Brain-derived neurotrophic factor (BDNF) is a member of the neurotrophin family of growth factors, capable of regulating neuronal development and viability. Thus, it may prevent neuronal apoptosis by inhibiting caspase-3 activation demonstrated extensively at a cellular level^4,5^. Many in vitro studies have proven that BDNF concentrations were higher in cancerous cells than in healthy ones^6–8^. The assessment of BDNF dependency could facilitate targeted tumor therapy. For therapeutic reasons, BDNF could be administered in vivo to a patient, for which a transporting carrier is required. However, its short half-life, prone to structural damage, resulting in rapid loss of its biological activity, limiting its application.

It should be underlined that BDNF’s role is rather complex. For instance, the literature data suggests that high BDNF levels might be associated with malignant disease progression and cancer cell survival^9,10^, and our previous studies showed that BDNF improves the recovery of damaged neuroblastoma cells^11^. On the other hand, high levels of proBDNF, an immature form of BDNF, are able to induce apoptosis^12–14^. This duality in BDNF’s role might be explained by the fact that BDNF can activate two types of receptors—mostly pro-survival Trk tyrosine kinase and p75 neurotrophin receptor (p75NTR), that contain a cytoplasmic death domain^15^. Depending on the cellular context, p75NTR can act pro-survival or activate apoptosis via the c-Jun N-terminal (JNK) pathway^16^.

It is worth noticing that the application of BDNF for therapeutic reasons also requires a thorough knowledge of its physicochemical properties, including its charge, stability as well as ability to attach to various biomaterials. However, despite essential significance, no systematic investigations were devoted to these issues. Only a few works were focused on the determination of charge of BDNF molecules. For example, the molecular mass of BDNF was determined to be 12.3 kDa with an isoelectric point ≥ 10.1^17^. Dąbkowska et al.^18^ found that BDNF molecules carry a net positive charge and adsorb onto negatively charged surfaces at pH 7.4.

Poly(diallyldimethylammonium chloride) (PDADMAC) is a synthetic, water-soluble polyelectrolyte with hydrophilic, positively charged quaternary ammonium group^19^. In biotechnology, it is applied in the formation of dendronized polymer gelator^20^, water treatment^21^, and dental material design^22^.

For a long time, heparin sodium salt (HP), being a natural, strongly charged polyanion, has been employed from conjugated to biodegradable and nondegradable synthetic polymers to enhance their biocompatibility^23^. In addition to its anti-thrombotic and anti-inflammation applications, novel uses for HP are being investigated^24^. The body easily absorbs similar biocompatible polysaccharides due to their interactions with proteins, growth factors, chemokines, cytokines, enzymes, and lipoproteins involved in various biological processes^25,26^. Additionally, it incorporates antiviral activity^27,28^, can control tumor growth, and is able to inhibit angiogenesis (possible cancer therapeutics^29,30^), as well as the complement cascade^31,32^.

A plethora of papers dedicated to the polyelectrolyte multilayers (films, PEMs) formation obtained by layer-by-layer (LbL) method^16^, was published within the last thirty years. Many thin polyelectrolyte films could potentially display biodegradability, are relatively straightforward to produce, and are expected to have a minimal impact on healthy cells while possibly exerting harmful effects on cancerous cells. In LbL technique, polydiallyldimethylammonium chloride (PDADMAC) is frequently used for the formation of anchoring layers^33^ and as the positively charged layers in building blocks^34^. However, in polyelectrolyte film preparation, PDADMAC is usually used with synthetic polyanions such as poly(styrene sulfonate) or poly(acrylic acid)^35^. Moreover, in order to assess the feasibility of constructing polyelectrolyte multilayers for medical applications, HP is mainly applied with chitosan, which serves as polycation^36^. It is also worth pointing out that bulk concentrations of the polyelectrolytes used for the formation of the multilayers in the LbL process are high and reach at least 1000–5000 mg/L^34,37^.

There are only a few papers where PDADMAC and HP are applied together in thin film formation. These films are frequently obtained by adsorption from high polyelectrolyte concentrations. For example, free-standing films were produced by mixing highly concentrated (0.125 M) PDADMAC and HP solutions^37^. PDADMAC/poly(styrene sulfonate) films with heparin, applied as the last layer of the coating, were prepared by adsorption from high bulk polyelectrolyte concentration, i.e., 5000 mg/L^38^. Recently, we have also published a paper devoted to controlled cellular adhesion on PDADMAC/HP films obtained from the low bulk polyelectrolyte concentration^39^. To the best of our knowledge, this is the first paper where the films were constructed by sequential adsorption of PDADMAC and HP from solutions of very low polyelectrolyte bulk concentration, i.e., 5 mg/L.

In light of the lack of sufficient information, the main aim of this research was to acquire essential physicochemical properties of the BDNF layers, adsorbed from very low bulk concentrations (1–5 mg/L) and formed on polyelectrolyte films composed of PDADMAC and HP. Accordingly, polyelectrolyte films, terminated either with negatively charged HP, referred to as “(PDADMAC/HP)_4_”, or with positively charged PDADMAC referred to as “(PDADMAC/HP)_3_/PDADMAC”, were used as effective carriers of the BDNF in a proliferation-limiting system for neuroblastoma cancer cells (referred to as “(PDADMAC/HP)_4_/BDNF” and “(PDADMAC/HP)_3_/PDADMAC/BDNF”, respectively).

Accordingly, the zeta potential of the BDNF layer covered polyelectrolyte films, the kinetics of BDNF adsorption on silica and the polyelectrolyte films, respectively, as well as the stabilities of BDNF layers were determined for the first time by applying various experimental techniques comprising inter alia the streaming potential, the optical waveguide lightmode spectroscopy (OWLS). The applied methods exhibit exceptional sensitivity; thus, reliable results for low BDNF and polyelectrolyte concentrations can be obtained. This knowledge was used to quantitatively determine the impact of the polyelectrolyte multilayers and polyelectrolyte coacervates, composed of PDADMAC, HP, and BDNF, on the protein-releasing profile, neuroblastoma cell viability and morphology, mitochondrial membrane potential, cell phenotype, and induction of lipid peroxidation. It should be pointed out that to the best of our knowledge, our study is the first paper explaining the impact of polyelectrolyte-based films formed from low macromolecule concentrations on neuroblastoma cancer cell morphology and viability.

## Materials and methods

All materials used in this study were analytical grade reagents and were used without further purification. Poly (diallyldimethylammonium chloride) (PDADMAC) of a molar mass of 101 kg/mol (number averaged) and 160 kg/mol (weight averaged) and heparin sodium salt (HP) for physicochemical analysis, employed as polycation and polyanion, respectively, were purchased from PSS Polymer Standards Service GmbH (Germany) and Merck (Germany).

Carrier-free recombinant human BDNF (248-BDB-250/CF), referred to as BDNF, was procured from R&D Systems (Canada). BDNF was dissolved in a PBS buffer and stored no longer than 48 h at a temperature of 4˚C.

Sodium chloride (NaCl) was supplied by Avantor Performance Materials Poland S.A. (formerly POCH S.A., Gliwice, Poland), and PBS fluid (balanced salt solution for laboratory use) was purchased from Biomed Lublin (Poland).

PDADMAC and HP powders were dissolved in NaCl solution with an ionic strength (*I*) of 0.01 M and pH 5.8, resulting in polyelectrolyte solutions with a constant bulk concentration of 5 mg/L before each adsorption experiment. The polyelectrolyte solutions were filtered through a disposable syringe filter of pore size 0.20 μm (Equimed, Poland) prior to use.

### Streaming potential measurements (SPM)

The zeta potentials of the successive polyelectrolyte layers, the BDNF layer, and the layer stabilities over time were determined through SPM. In the experiments, we utilized a homemade streaming potential (SP) cell, which was described in detail in Refs.^40,41^. The SP cell consisted of two polished Teflon blocks having two inlet and outlet compartments. Two silicon-covered silica (Si/SiO_2_) plates were positioned on the blocks, separated by a Teflon gasket serving as a spacer. The silicon wafers (plates) were commercially sourced from Siegert Wafer GmbH, Germany, and were used as substrates for polyelectrolyte multilayer formation in streaming potential measurements (SPM).

The silicon plates were cleaned by immersing them for 30 min in piranha solution, which is a mixture (1:1 ratio) of 95% sulfuric acid and 30% hydrogen peroxide. After cleaning, the wafers were thoroughly washed with deionized water and immersed in the 80 ºC water for 30 min. Wafers prepared in this manner were stored in ultrapure water for no longer than 48 h.

The parallel plate channel was formed by clamping the blocks together with two Si/SiO_2_ plates and the spacer, using a press under constant torque conditions. The entire system was placed inside the earthen Faraday cage to avoid any disturbances stemming from external electric fields. The streaming potential *ΔE*_*s*_, which occurs when a pure electrolyte flows through the SP cell underregulated and constant hydrostatic pressure difference *ΔP*, was measured using the two Ag/AgCl electrodes. The use of a high resistance electrometer (Keithley 6512) allowed for performing the potential measurements under practically zero current conditions.

A series of streaming potential measurements were conducted at four various pressures to induce flow through the cell. This allowed for obtaining the slope of the dependence *ΔE*_*s*_ vs. *ΔP*, which is necessary for determining the zeta potentials of the subsequent layers using the Smoluchowski equation^42^.

The experimental procedure for evaluating the zeta potential consisted of measuring the zeta potential of Si/SiO_2_, forming the desired number of polyelectrolyte layers (seven and eight layers with PDADMAC and HP on top, respectively) on Si/SiO_2_ and adsorbing of BDNF either on (PDADMAC/HP)_3_/PDADMAC or (PDADMAC/HP)_4_ films. After adsorbing each layer, the *ΔE*_*s*_ vs. *ΔP* dependence was determined, and the zeta potential was calculated using the Smoluchowski formula.

The desorption of the polyelectrolyte chains and BDNF molecules from the multilayers was also studied. After completing the desired number of layers, terminated either by PDADMAC, HP, or BDNF, the zeta potential of the multilayer was assessed at specified time intervals (usually every 30 min).

The initial bulk concentration of the polyelectrolytes (PDADMAC and HP) was 5 mg/L, while BDNF was adsorbed from the solution of 0.1 and 1 mg/L, respectively. The pH and ionic strength of the polyelectrolyte solutions were defined and set at 5.8 and 0.01 M NaCl, respectively. BDNF was adsorbed from PBS fluid with an ionic strength of 0.15 M and pH 7.4. The adsorption process occurred inside the SP cell for a fixed period (20 and 25 min for polyelectrolytes and BDNF, respectively) under flow-controlled transport conditions (flow velocity, *V*_flow_, was 0.02 ml s^-1^ for the polyelectrolytes and 0.01 ml/s for BDNF)*.* The adsorbed polyelectrolyte films were rinsed with pure PBS fluid before BDNF adsorption. After BDNF adsorption, the BDNF-coated polyelectrolyte films were also flushed with pure PBS.

### Optical waveguide lightmode spectroscopy (OWLS)

The OWLS sensors (MicroVacuum Ltd., Hungary) are made of glass support (with a refractive index *n*_*S*_ = 1.52578) coated with 170 nm Si_0.78_Ti_0.22_O_2_ (refractive index *n*_*F*_ = 1.8), and an additional 10 nm layer of pure SiO_2_ were applied in the optical waveguide lightmode spectroscopy (OWLS).

The OWLS sensor chips were thoroughly cleaned prior to each experiment. The sensors were sonicated in Hellmanex solution (3%) within an ultrasound bath for 15 min. Subsequently, they were rinsed and sonicated multiple times with Milli-Q water. After rinsing, the sensors were dried with nitrogen flow and placed in a UV cleaner for 15 min. They were then rinsed again with Milli-Q water and dried with a gentle stream of nitrogen. The sensors were used immediately after the cleaning process.

Real-time measurements of the surface mass densities of adsorbed polyelectrolytes and BDNF were performed using OWLS apparatus. OWLS detects refractive index changes 100–200 nm above the sensor’s surface, providing quantitative information on near-surface kinetic processes.

The adsorption onto the waveguide surface leads to alterations in the interfacial refractive index, which, in turn, affect the incoupling angles of the laser light, and these changes are monitored. Assuming an optically uniform adsorbed layer, the mass of adsorbed polyelectrolytes or proteins is calculated using Feijter’s formula^43^

1 $$\Delta m_{OWLS} = l_{adlayer} \frac{{n_{adlayer} - n_{solusion} }}{{\left( {dn_{adsorbate} /dc_{adsorbate} } \right)}}$$

where *l*_*adlayer*_ (cm) and *n*_*adlayer*_ are the thickness and the refractive index of the adlayer, respectively, *dn*_*adsorbate*_*/dc*_*adsorbate*_ is the refractive index increment of the respective adsorbate. *dn*_*adsorbate*_*/dc*_*adsorbate*_ equal 0.18 cm^3^/g and 0.15 cm^3^/g for the polyelectrolytes, and proteins, respectively. *n*_*solution*_ is the refractive index of the solutions.

In contrast to gravimetric methods, the OWLS technique provides the dry mass of adsorbed molecules. A standard in situ OWLS experiment commenced with the flow of pure electrolyte solution to condition the surface and establish a stable baseline (*Δm* < 15 ng/cm^2^ per 1 h). As the silica-covered sensor is negatively charged^44^, the first polyelectrolyte layer was formed by supplying a solution of positively charged PDADMAC solution over the sensor surface, leading to a signal shift. Upon adsorption onto the sensor’s surface caused a shift in effective refractive indexes towards higher values, enabling the in situ monitoring of adsorption kinetics. After a rinsing step with electrolyte solution to remove the loosely bound molecules from the surface, the measuring cell was filled with negatively charged HP solution, and the kinetics of HP adsorption were monitored. The experiment ended with a rinsing phase. After achieving a stable final signal, the entire procedure was repeated 3–4 times to obtain the desired number of layers (6–8). Finally, BDNF was adsorbed onto the polyelectrolyte layers, and the resulting film was rinsed with a pure electrolyte solution for at least 50 min. It should be noted that each layer (PDADMAC, HP, BDNF) was washed with pure electrolyte during the formation of the coating.

### Cell culture

The SH-SY5Y neuroblastoma cell line (human, ECACC; Sigma Aldrich, St. Louis, MO, USA) was cultured using a 1:1 mixture of Ham’s F-12 Nutrient Mixture (Thermo Fisher, Waltham, MA, USA) and Minimum Essential Medium (MEM) (Sigma Aldrich, St. Louis, MO, USA). This culture medium was supplemented with streptomycin (100 U/mL), penicillin (100 µg/mL), l-glutamine (2 mM), and 10% heat-inactivated fetal bovine serum (FBS) (all reagents are Gibco, Thermo Fisher Scientific, Waltham, MA, USA), and it is referred to as “proliferation medium” in text. Reduced serum (1% FBS) differentiation medium was used in the experiments to slow down the proliferation and allow for eventual cell differentiation. The cells were maintained at 37 °C in an environment with saturated humidity and 5% CO_2_. The proliferation medium was changed every 2–3 days, and the cell passageing was performed once they reached 80% confluence.

### Preparation of PEMs for in vitro studies

PEMs, consisting of positively charged PDADMAC and negatively charged HP layers, were employed as carriers of BDNF molecules. Before adsorption, polyelectrolyte solutions (1000 mg/L, 0.01 M NaCl, pH 5.8) were diluted to a concentration of 5 mg/L using 0.01 M NaCl, pH 5.8. A 96-well plate (Thermo Fisher Scientific, Waltham, MA, USA) was coated with either 7 or 8 layers of polyelectrolytes using the LbL method. The adsorption time of both PDADMAC and HP was 20 min. Following After each adsorption step, the wells were rinsed three times with 0.01 M NaCl, at pH 5.8. This process was repeated until the desired number of layers was obtained. Finally, the wells were washed three times with PBS fluid (*I* = 0.15 M, pH 7.40).

### BDNF adsorption on PEM

Stock solutions of BDNF were prepared at concentrations of 1 mg per liter (1 mg/L) and 0.1 mg per liter (0.1 mg/L) were prepared in PBS shortly before being used for adsorption onto polyelectrolyte multilayers. The adsorption of BDNF onto the PEM was carried out at room temperature for 15 min. Subsequently, the BDNF-coated polyelectrolyte films underwent three rinses with PBS. After this preparation step, SH-SY5Y cells were seeded onto the BDNF-coated surfaces.

### DuoSet ELISA test

All reagents for the DuoSet ELISA test were purchased from R&D Systems (Canada). The protein release profile from PEMs in bulk/in vitro, residual BDNF concentration, and cellular uptake were determined utilizing ELISA DuoSet assay (DY248) according to the manufacturer’s instructions.

Briefly, polystyrene plates (DY990) were covered by ELISA coating buffer (DY006) containing diluted capture antibody (part of the DuoSet kit). After an overnight incubation, the plate was washed three times with a washing buffer (WA126). Then, wells were blocked with Reagent Diluent (DY995) for 1 h, and samples as well as standards were added. The next steps involved the addition of detection antibody diluted in Reagent Diluent (part of the DuoSet kit), Streptavidin-HRP (DY998), and Substrate (DY999), respectively, with washing steps in between and incubation times as suggested in the datasheet. All the incubations were at room temperature. Finally, Stop Solution (DY994) was added, and the absorbance was immediately read at λ_450_ and λ_540_ (as a wavelength correction) with Varioskan™ LUX multimode microplate reader (Thermo Fisher Scientific, Waltham, MA, USA). SkanIt™ Software for Microplate Readers ver. 5.0 was used for the analysis of the data.

### Cell viability

For the cell viability assessment, SH-SY5Y cells were seeded at a density of 3 × 10^4^ cells per well in a 96-well plate in the following experimental conditions: control (no layers, no BDNF), BDNF (0.1 mg/L or 1 mg/L), (PDADMAC/HP)_3_/PDADMAC, (PDADMAC/HP)_3_/PDADMAC/BDNF (1 mg/L), (PDADMAC/HP)_3_/PDADMAC/BDNF (0.1 mg/L), (PDADMAC/HP)_4_, (PDADMAC/HP)_4_/BDNF (1 mg/L), (PDADMAC/HP)_4_/BDNF (0.1 mg/L) layers. During adherence, cells were incubated for 2 h in a reduced serum differentiation medium. After an additional 24 h, the viability of neuroblastoma cells was assessed using the Alamar Blue® reagent assay (Thermo Fisher Scientific, Waltham, MA, USA) according to the manufacturer's instruction. The Alamar Blue® assay is based on the ability of healthy cells to convert resazurin into fluorescent resorufin. The level of generated fluorescence is indicative of the cell population’s viability. The fluorescence readings were obtained using a Varioskan™ LUX multimode microplate reader (Thermo Fisher Scientific, Waltham, MA, USA). SkanIt™ Software for Microplate Readers ver. 5.0 was used for the analysis of the data.

### BDNF bulk and in vitro concentration

To determine the concentration of BDNF, supernatants (medium with 1% FBS serum) from SH-SY5Y cells incubated with PEMs were collected after 24 h. This process was carried out with at least six replicates for each experimental variant. The BDNF concentration in these supernatants was measured with Duoset ELISA test.

### Detection of mitochondrial membrane potential deterioration

JC1 is a marker of mitochondrial wellbeing. In a healthy cell with high mitochondrial membrane potential, JC-1 forms complexes emitting red fluorescence (J-aggregates). In an apoptotic or unhealthy cell with low mitochondrial membrane potential, the dye remains in its monomeric form emitting green fluorescence (J-monomers). SH-SY5Y cells were seeded in a 96-well black plate with clear bottom, coated with multilayers (as described in ‘Preparation of PEMs for in vitro studies’) and BDNF protein in either of the two concentrations (1 mg/L or 0.1 mg/L, respectively). Cells were incubated for 24 h. Subsequently, according to the manufacturer’s instructions, cells were stained with JC-1 Mitochondrial Membrane Potential Assay Kit (Cayman Chemical Company, Ann Arbor, MI, USA) for 30 min at 37 °C in a CO_2_ incubator. Fluorescence was measured at Ex535nm/Em595nm and Ex485nm/Em535nm using Varioskan™ LUX multimode microplate reader (Thermo Fisher Scientific, Waltham, MA, USA). Subsequently, ratios between J-aggregates to J-monomers of each sample were calculated and presented in relation to the control well.

### Detection of lipid peroxidation

Lipid peroxidation is mainly caused by free radical attacks on specific compounds such as polyunsaturated fatty acids (oxidative stress). The malondialdehyde (MDA) concentration in cell cultures can be used to assess the extent of this process.

After adhesion to the plate, SH-SY5Y cells (3 × 10^4^ cells/well) were incubated on multilayers (described in “Preparation of PEMs for in vitro studies”) for 24 h. Experimental details were indicated in previous sections. SH-SY5Y cells treated with 1000 μM H_2_O_2_ were included as an oxidative stress control. After the incubation period, the cell supernatant was collected from each well. The MDA concentration in the cell supernatant was determined using reverse-phase, high-performance liquid chromatography (HPLC)-spectrophotometric method. This method utilized (Agilent 1260 Infinity II HPLC system, Agilent Technologies, Waldbronn, Germany)^45^ and BDS Hypersil C18 column. Freshly prepared 10 mM TEP (1,1,3,3-tetraethoxypropane) was diluted in water to create working calibrants at concentrations 0.05, 0.075, 0.1, 0.15, and 0.2 µM TEP. 100 µL of the supernatant or standard was treated with 200 µL of 5% TCA (trichloroacetic acid) and 10 µL of 0.4% BHT (butylated hydroxytoluene) in absolute ethanol for deproteinization and antioxidant effect. The samples were vortexed. Subsequently, 100 µL of 0.6% TBA (2-thiobarbituric acid) was added and then reacted for 45 min at 90 °C to form an adduct MDA-(TBA)_2_, which can be detected at 532 nm. After the final centrifuge at 8000 × *g* (10 min, 4 °C) supernatants were collected and adjusted to pH 7.0 with 1 M NaOH. The elution buffer was determined to be 50 mM KH_2_PO_4_ (pH 7.0 fixed with KOH) and CH_3_OH (70:30, v/v). The sample run was 6.5 min, with a flow rate of 0.5 mL/min, an injection volume of 20 µL, and visible excitation and emission at 528 and 553 nm. The analysis of MDA release by the human neuroblastoma cells SH-SY5Y was performed by integrating the retention times and the peak areas compared with known concentrations of MDA calculated as mM MDA equivalent from the TEP standard calibration (1:1 conversion under acidic conditions).

### Protein lysate isolation

SH-SY5Y cells were seeded as previously described in the upper sections. After 24 h of incubation, cells were washed with 1 × PBS (P4417, Sigma Aldrich, St. Louis, Missouri, United States), trypsinized (#25200056, Thermo Fisher Scientfic, Waltham, MA, USA) for 5 min at 37 °C, followed by centrifugation at 1000 × *g* for 5 min. Pelleted cells were resuspended in the cell lysing mixture of 1 × RIPA Lysis and Extraction Buffer, 1 × Halt™ Protease Inhibitor Cocktail and 1 × Halt™ Phosphatase Inhibitor Cocktail (Cat. No. 89901, 87786 and 78420 respectively, Thermo Fisher Scientfic, Waltham, MA, USA). After 20 min. of incubation at 4 °C, the remains of the cells were centrifuged at 15,000 × *g* in 4 °C for 15 min. Lysates were immediately transferred to − 80 °C for future analysis.

### BDNF uptake in SH-SY5Y

Total protein concentration of the collected cell lysates (n = 6) was quantified with a BCA protein assay kit for low concentrations (ab207002, Abcam, Cambridge, United Kingdom), according to the manufacturer’s protocol. Briefly, whole cell lysates with unknown protein concentration, and reference protein standard (bovine serum albumin, concentration range 0.5–40 μg/mL) were incubated with green-colored BCA Working Solution for two hours at 37 °C. The total protein concentration was then determined by measuring the color change of the sample solution from green to purple, which is proportional to the protein concentration. Absorbance was measured against λ_562_ using Varioskan™ LUX multimode microplate reader (Thermo Fisher Scientific, Waltham, MA, USA). SkanIt™ Software for Microplate Readers ver. 5.0 was used for the analysis of the data.

BDNF concentration in 1 µg of the whole cell lysate was subsequently determined using DuoSet ELISA test.

### The analysis of cell morphology

Cells were seeded as described in previous sections. After 24 h, the medium was changed to a fresh portion of the differentiation medium (as described in “Cell Culture”). The cells were further cultured until 7 days after seeding. The medium was refreshed every 2–3 days. After 8 days, pictures were taken with a Progres Gryphax BETRIA camera (Jenoptic) from under the Leica DMIL LED microscope at 10×/0.25 PH1 and 20×/0.35 PH1(Leica) magnification.

### Statistical analysis

The data in this study are presented as means ± standard deviations (SD) and are based on a minimum of three independent experiments. The Kruskal–Wallis test and one-way ANOVA followed by Dunnett’s multiple comparisons test, performed using GraphPad Prism (version 9.1.1 for Windows, GraphPad Software, San Diego, California USA, www.graphpad.com) were used to do statistical analysis between each study group. For analysis comparing experimental groups, a two-way ANOVA was utilized. Statistics were judged significant at *p < 0.05, **p < 0.01, ***p < 0.001.

### Ethics approval and consent to participate

This work isn’t involved in the animal protocols approved by the Research Animal Care and Use Committee. SH-SY5Y cells were purchased from Sigma Aldrich, St. Louis, MO, USA.

## Results

### Physicochemical characterization of BDNF-loaded PEM

#### SPM

Polyelectrolyte adsorption is mainly driven by electrostatic forces, but other surface-protein interactions like van der Waals, hydrophobic, and hydrogen bonding also play a vivid role in protein adsorption^46–48^. Thus, the knowledge of successive layer charges is important for the proper interpretation of polyelectrolyte/ BDNF film formation.

Reliable measurements of the subsequent layer charge (the zeta potential) could be performed with SPM. It provides the signal from a large area corresponding to the channel size and it is sensitive only to the charge of the outermost layer. Accordingly, the average value of the zeta potential of the outermost layers of the film can be easily determined.

**Figure 1:**
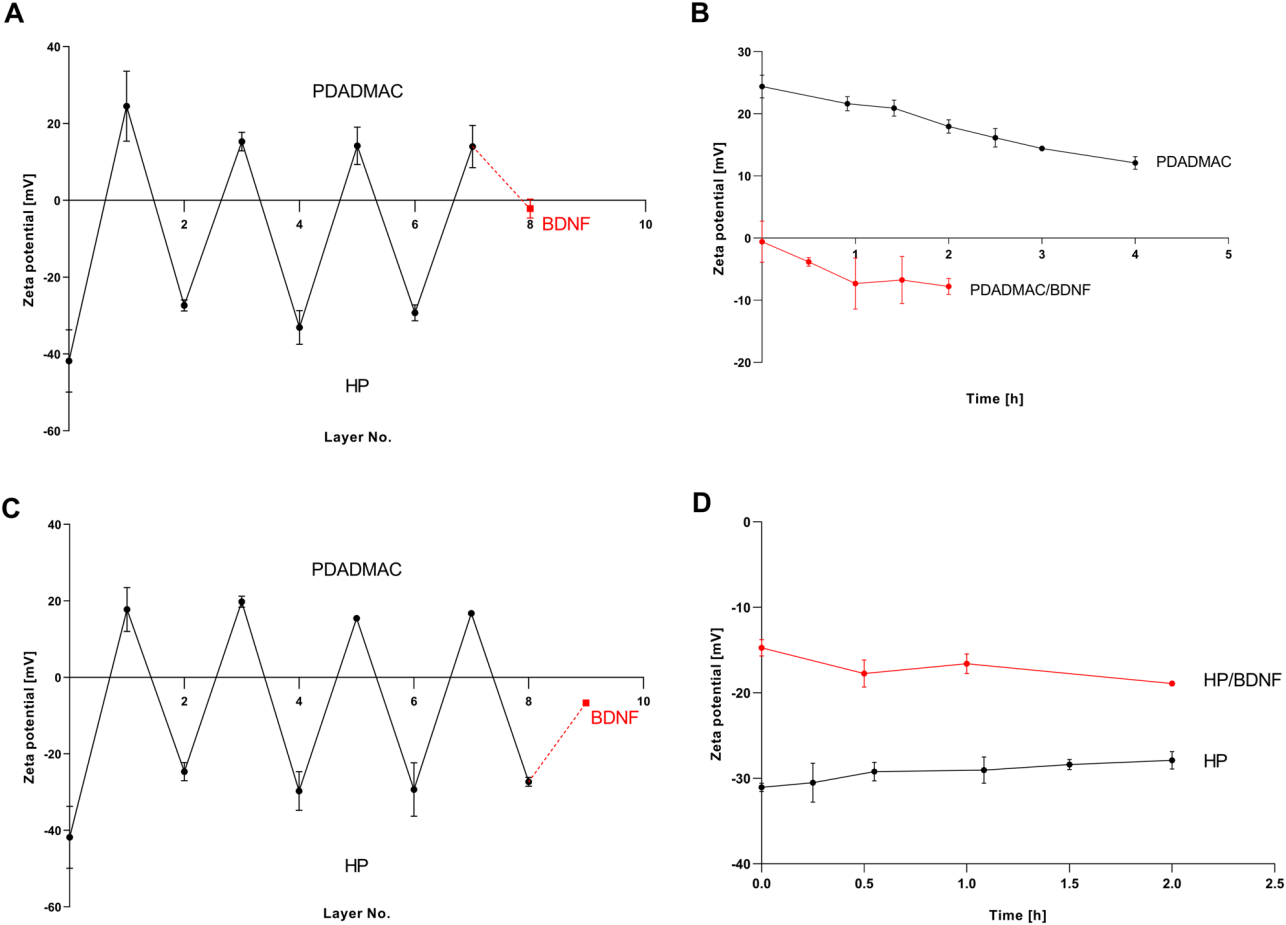
The zeta potential of Si/SiO_2_ as the function of the number of adsorbed layers determined for (PDADMAC/HP)_3_/PDADMAC supplemented by BDNF (**A**); The stabilities of (PDADMAC/HP)_3_/PDADMAC and BDNF adsorbed on (PDADMAC/HP)_3_/PDADMAC (**B**); The zeta potential of Si/SiO_2_ as the function of the number of adsorbed layers determined for the (PDADMAC/HP)_4_ supplemented by BDNF (**C**); The stabilities of: (PDADMAC/HP)_4_ and BDNF adsorbed on (PDADMAC/HP)_4_ (**D**). Polyelectrolyte adsorption conditions: 5 mg/L, *I* = 0.01 M, pH = 5.8, adsorption time 20 min. BDNF was adsorbed from the bulk concentration of 1 mg/L and *I* = 0.15 M, pH 7.4 for 25 min. The points denote SPM results obtained in the NaCl of pH 5.8, 0.01 M NaCl (polyelectrolytes) and in PBS of pH 7.4, 0.15 M (BDNF). Lines represent the fit of experimental data.

The formation of PEM composed of PDADMAC and HP on Si/SiO_2_ was achieved through the sequential adsorption of the polyelectrolytes. When the zeta potential of each successive layer was plotted against the layer number, zig-zac curves were obtained, as presented in Fig. 1A and C.

After evaluation of the zeta potential of each layer forming PEM, the channel was rinsed with pure PBS fluid. Subsequently, the BDNF solution with a bulk concentration of 1 mg/L was introduced into the cell, and the zeta potential of the multilayer was determined.

To assess the potential of using the PDADMAC/HP multilayers as an effective carrier for BDNF delivery, the stabilities of PDADMAC- terminated and HP-terminated multilayers, as well as the BDNF-supplemented multilayers were also examined (as shown in Fig. 1B and D). The PEM stabilities were monitored under stationary conditions, and the zeta potentials for these layers were evaluated at defined intervals.

By examining Fig. 1A and C, one can observe that the adsorption of the successive layers leads to periodic changes in zeta potential, including zeta potential inversion. BDNF supplementation resulted in changes in both the sign and value of the zeta potential of the PDADMAC-terminated layer, shifting it from 14 to − 2 mV. Conversely, the zeta potential of HP-terminated layer increased from − 27 to − 6 mV. This indicated the successful adsorption of BDNF on both types of multilayers.

As shown in Fig. 1B, the zeta potential of the PDADMAC-terminated film remained stable for 1.5 h of washing, with only a decrease from 24 to 12 mV, observed after 4 h. The changes in zeta potentials of the (PDADMAC/HP)_4_ multilayers over a 2-h washing period were practically negligible, as seen in Fig. 1D. These results suggest that both PDADMAC- terminated and HP-terminated thin films are stable for at least 2 h, making them suitable for use as carriers for BDNF.

The BDNF stabilities on (PDADMAC/HP)_3_/PDADMAC and (PDADMAC/HP)_4_ films were presented in Fig. 1B and D. The zeta potential of the BDNF-covered PDADMAC-terminated multilayers decreased slightly within the first hour, shifting from − 1 to − 7 mV. However, no significant changes in zeta potential were observed when BDNF was adsorbed on HP-terminated multilayers within 2 h.

#### OWLS

**Figure 2:**
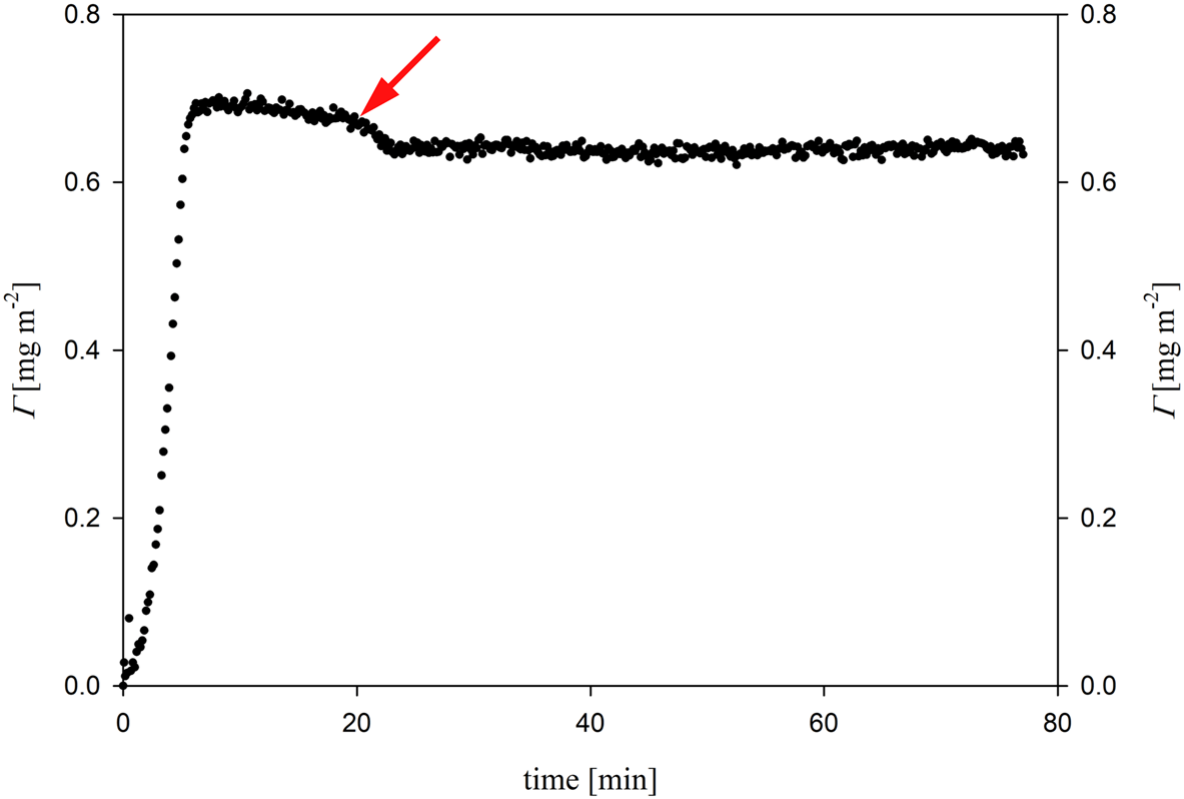
Kinetics of BDNF adsorption on silica expressed as the dependence of the coverage (*Γ*) on time. Bulk protein concentration was 5 mg/L, pH 5.6, NaCl concentration was 0.15 M, flow rate 2.5 × 10^–3^ cm^3^/s. The arrow shows the beginning of the desorption run.

The initial series of OWLS measurements was devoted to the determination of BDNF adsorption/desorption kinetic on the bare silica surface. A typical kinetic run, performed at pH 5.6, NaCl concentration of 0.01 M, and a bulk protein concentration of 5 mg/L, is shown in Fig. 2. The data clearly illustrate the effective adsorption of BDNF on negatively charged OWLS sensors. For BDNF coverages below 0.6 mg/m^2^, the adsorption kinetic remains a linear function of time. After 10 min of adsorption, the kinetics attained a limiting coverage of ∼ 0.7 mg/m^2^. Subsequently, after 20 min of adsorption, the desorption experiment was initiated. In this phase, a pure electrolyte having the same ionic strength and pH, was flushed through the cell at the same flow rate. It's notable that the amount of desorbed BDNF was relatively limited, amounting to ∼ 0.05 mg/m^2^ over the course of the desorption run that lasted 60 min.

In the next step, the kinetics of BDNF adsorption on both (PDADMAC/HP)_3_/PDADMAC and (PDADMAC/HP)_4_ films were determined.

**Figure 3:**
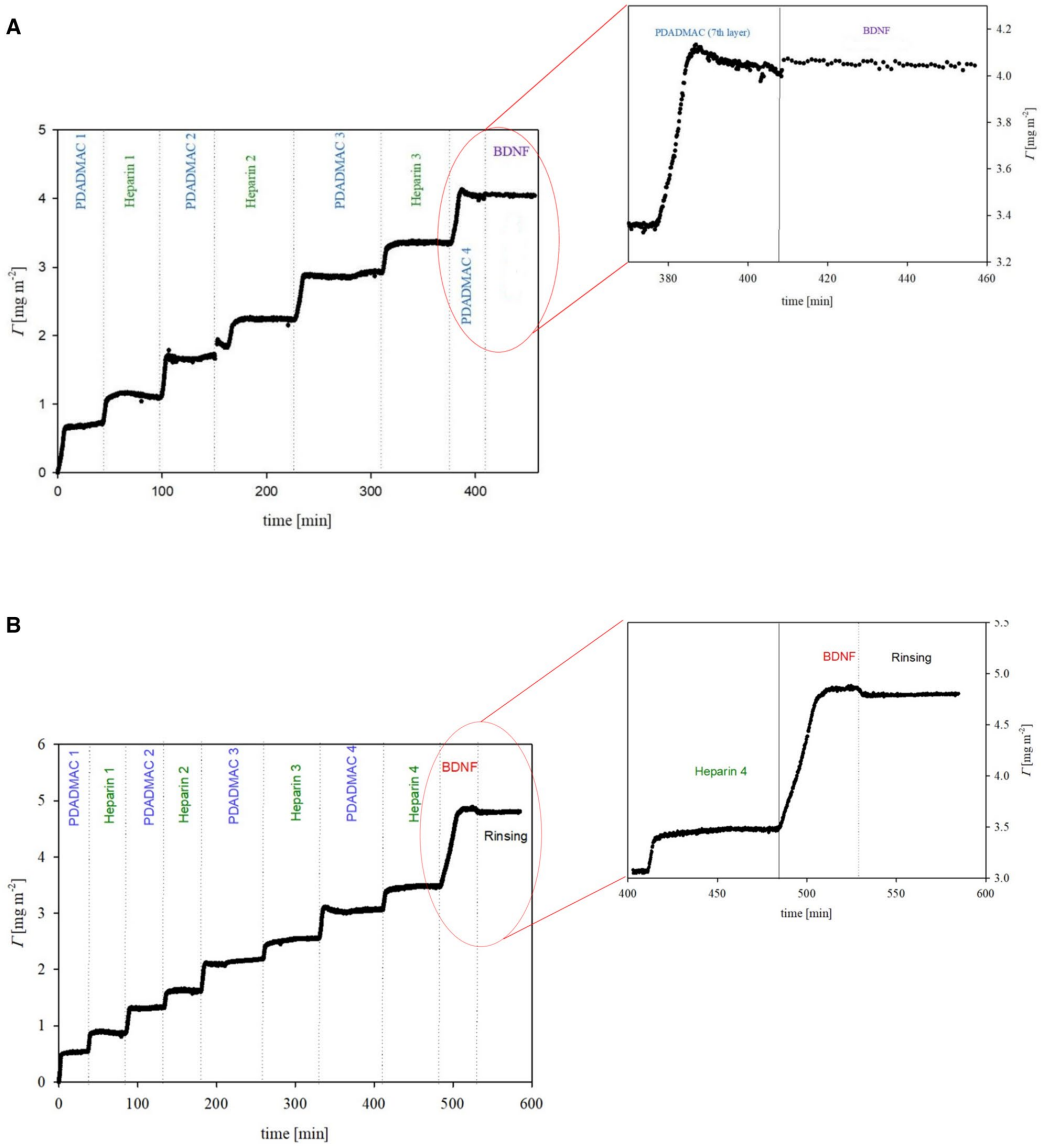
Kinetics of BDNF adsorption on PEM, determined by OWLS, expressed as the dependence of the coverage (*Γ*) on the time. Bulk protein concentration was 1 mg/L, pH 5.6, NaCl concentration: 0.15 M, and flow rate: 2.5 × 10^–3^ cm^3^/s. Part (**A**) BDNF adsorption on (PDADMAC/HP)_3_/PDADMAC, part (**B**) BDNF adsorption on (PDADMAC/HP)_4_.

Figure 3 depicts the BDNF adsorption kinetics on (PDADMAC/heparin) multilayers preadsorbed on OWLS sensor. As seen can in Fig. 3A, BDNF either did not adsorb onto the positively charged (PDADMAC/HP)_3_/PDADMAC, or its adsorption is reversible. There is no observable increase in mass under these conditions, likely due to electrostatic repulsion between the slightly positively charged protein and the PEM that terminates with the positively charged PDADMAC layer. On the other hand, for the (PDADMAC/HP)_4_ multilayer (as shown in Fig. 3B), efficient BDNF adsorption is evident. The initial kinetic of BDNF adsorption remains linear for short adsorption times. After reaching a stable plateau value, negligible desorption of BDNF is observed. This unequivocally indicates the successful adsorption of BDNF on negatively charged surfaces such as silica and HP-terminated PEMs. During the study of the process of formation of multilayer systems composed of PDADMAC and heparin, followed by BDNF adsorption using the OWLS method, the thicknesses of the formed films were also monitored. These thicknesses equal 1 and 6 nm for the BDNF layer on a bare silica sensor and (PDADMAC/HEPARIN)_3_BDNF, respectively (Supporting Information Figs. 1 and 2).

### Cell viability study

The impact of PEM and BDNF on neuroblastoma viability was studied using Alamar Blue® assay. SH-SY5Y cells were cultured for 24 h on (PDADMAC/HP)_3_/PDADMAC or (PDADMAC/HP)_4_ multilayer, with or without adsorbed BDNF (0.1 mg/L or 1 mg/L). As a control, cells were not exposed to BDNF and/or multilayers.

**Figure 4:**
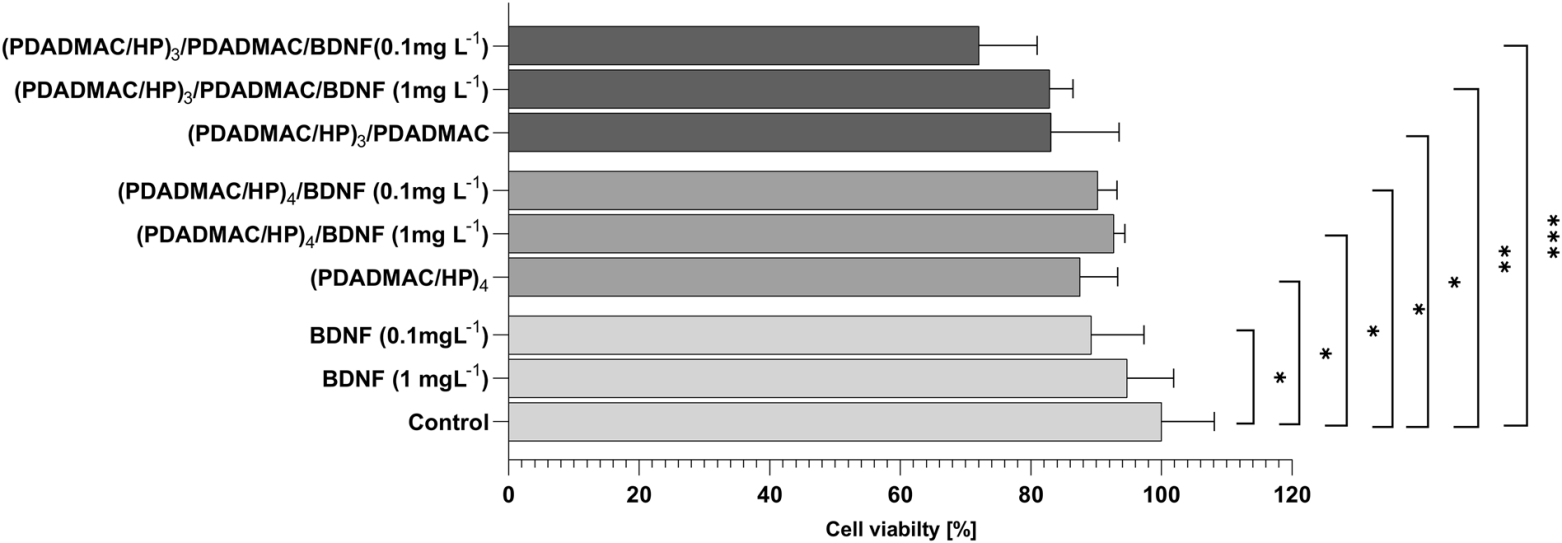
Effect of BDNF, PDADMAC/HP, and PDADMAC/HP/BDNF multilayers on cell viability. SH-SY5Y cell line was exposed to various concentrations of BDNF (0.1 mg/L or 1 mg/L), (PDADMAC/HP)_3_/PDADMAC, (PDADMAC/HP)_3_/PDADMAC/BDNF (1 mg/L) (PDADMAC/HP)_3_/PDADMAC/BDNF (0.1 mg/L), (PDADMAC/HP)_4_, (PDADMAC/HP)_4_/BDNF (1 mg/L), (PDADMAC/HP)_4_/BDNF (0.1 mg/L) layers for up to 24 h. As a control, cells were not exposed to BDNF and/or multilayers, (A) Column graph showing mean ± SD of cell viability in relation to control in each setting. For a detailed table containing the statistical significance of tested variables, see Supporting Information Table 1. Statistical analysis was determined using Kruskal–Wallis, one-way ANOVA and Two-Way ANOVA: ****p < 0.0001, ***p < 0.001, ** < 0.01, *p < 0.05.

We observed a significant reduction in viability when cells were cultured on (PDADMAC/HP)_3_/PDADMAC (83.1% ± 10.4%) and (PDADMAC/HP)_4_ (87.5% ± 5.7%) multilayers (see Fig. 4 and Supporting Information Table 1). Contact with BDNF adsorbed on (PDADMAC/HP)_3_/PDADMAC multilayer, further decreased their viability to 72.1% ± 8.9% and 82.9% ± 3.5%, respectively. The cell viability was generally lower when they were cultured on the PDADMAC-terminated multilayer.

### Mitochondrial membrane potential study

**Figure 5:**
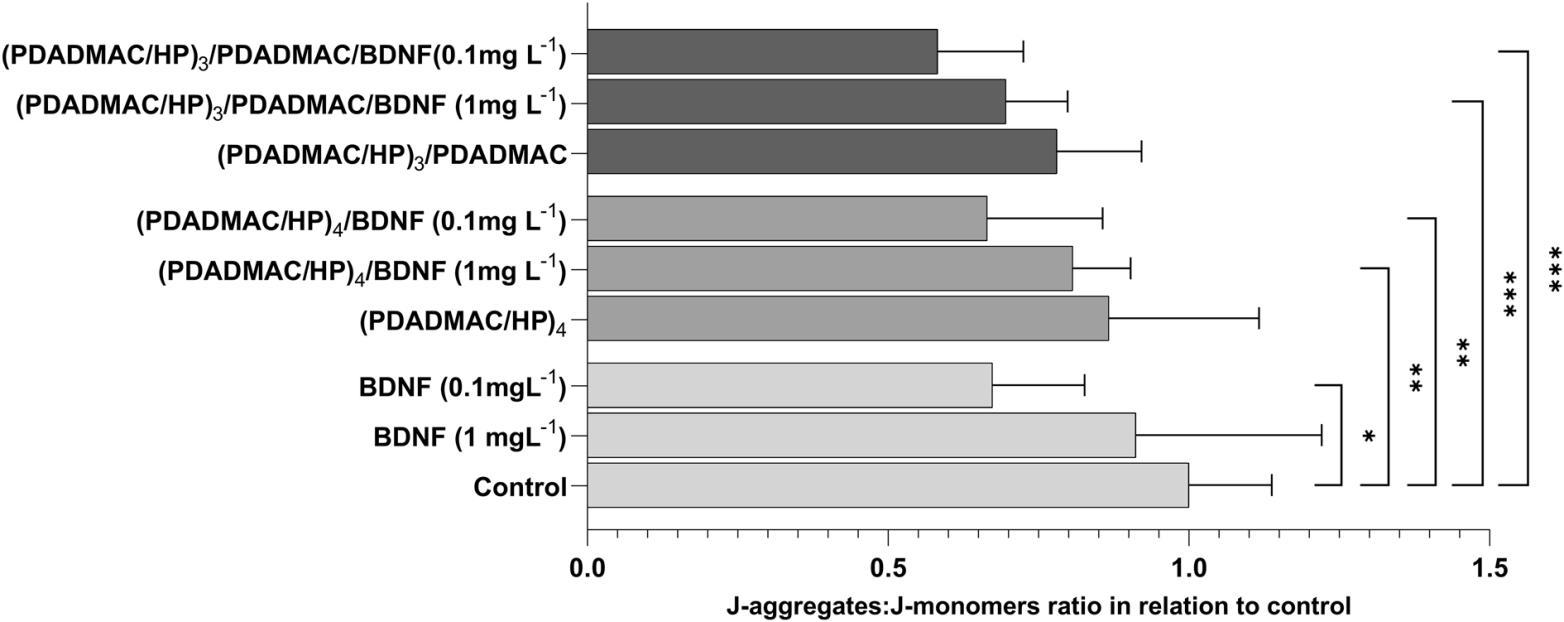
Effect of BDNF, PDADMAC/HP, and PDADMAC/HP/BDNF multilayers on mitochondrial membrane potential. The SH-SY5Y cell line was exposed to various concentrations of BDNF (0.1 mg/L or 1 mg/L), (PDADMAC/HP)_3_/PDADMAC, (PDADMAC/HP)_3_/PDADMAC/BDNF (1 mg/L), (PDADMAC/HP)_3_/PDADMAC/BDNF (0.1 mg/L), (PDADMAC/HP)_4_, (PDADMAC/HP)_4_/BDNF (1 mg/L), (PDADMAC/HP)_4_/BDNF (0.1 mg/L) layers for up to 24 h. As a control, cells were not exposed to BDNF and/or multilayers. JC-1 fluorescence ratio was calculated as the J-aggregates to J-monomers ratio of setting in relation to the J-aggregates to J-monomers ratio of the control. Column graph showing mean in relation to control ± SD of JC-1 fluorescence ratio in relation to control in each setting. For a detailed table containing the statistical significance of tested variables, see Supporting Information Table 2. Statistical analysis was determined using Kruskal–Wallis, one-way ANOVA, and Two-Way ANOVA: ****p < 0.0001, ***p < 0.001, ** < 0.01, *p < 0.05.

Mitochondrial membrane potential is a consecutive parameter allowing us to deepen our knowledge of the condition of cells grown in each setting. SH-SY5Y cell line was cultured on the BDNF-covered (PDADMAC/HP)_3_/PDADMAC or (PDADMAC/HP)_4_ layer. As a control, cells were not exposed to BDNF and/or multilayers. After 24 h, we analyzed their health by adding JC-1 dye to the cell culture as described in materials and methods. The results were in agreement with previous findings. Both the (PDADMAC/HP)_4_ and PDADMAC/HP)_3_/PDADMAC resulted in a decrease in cell viability (drop by 13% and by 22%, respectively) (Fig. 5 and Supporting Information Table 2). BDNF adsorption further worsened mitochondrial membrane potential. Particularly, potential decreased by 34% and by 42%, respectively, on the (PDADMAC/HP)_4_/BDNF (0.1 mg/L) and on the (PDADMAC/HP)_3_/PDADMAC/BDNF (0.1 mg/L) multilayer.

### BDNF release (in vitro in SH-SY5Y cell line and in bulk-culture medium)

**Figure 6:**
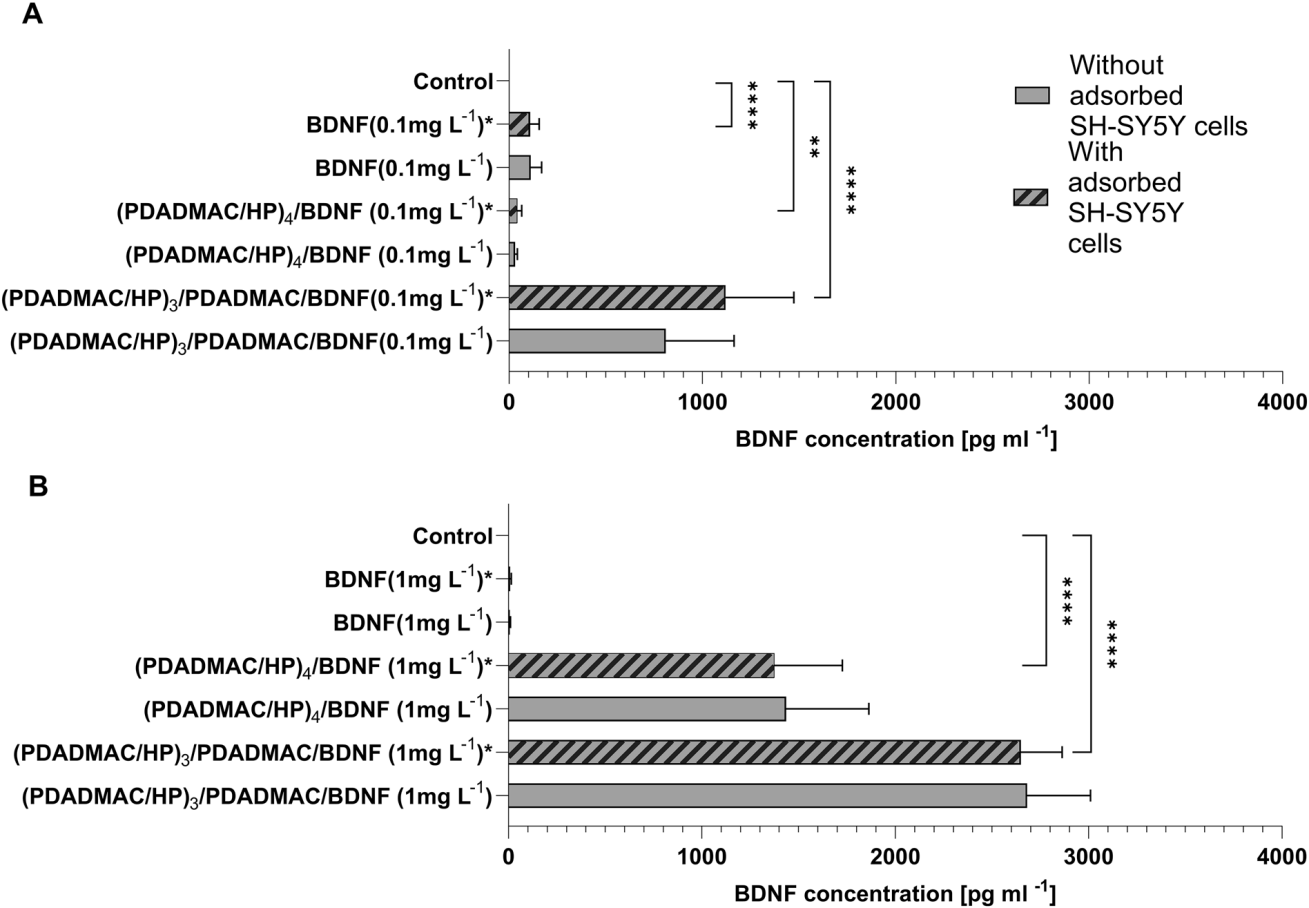
Effect of BDNF, PDADMAC/HP, and PDADMAC/HP/BDNF multilayers on BDNF release to medium. SH-SY5Y cell line was exposed to various concentrations of BDNF (0.1 mg/L or 1 mg/L), (PDADMAC/HP)_3_/PDADMAC, PDADMAC/HP)_3_/PDADMAC/BDNF (1 mg/L), PDADMAC/HP)_3_/PDADMAC/BDNF (0.1 mg/L), (PDADMAC/HP)_4_, (PDADMAC/HP)_4_/BDNF (1 mg/L), (PDADMAC/HP)_4_/BDNF (0.1 mg/L) layers with or without adhered SH-SY5Y cells for up to 24 h. (**A**) Column graph showing mean ± SD of BDNF concentration (pg/mL) in each setting. For a detailed table containing the statistical significance of tested variables, see Supporting Information Table 3. Data is presented as the mean ± SD (n = 3). Statistical analysis was determined using Kruskal–Wallis, one-way ANOVA, and Two-Way ANOVA: ****p < 0.0001, ***p < 0.001, ** < 0.01, *p < 0.05.

Further, we sought to investigate the levels of BDNF released into the cell culture medium in relation to polyelectrolyte (PDADMAC or HP-terminated) multilayer and/or concentrations of adsorbed BDNF molecules (0.1 mg/L or 1 mg/L) with the same settings as while studying cell viability. As expected, we observed that both BDNF-terminated multilayers caused a concentration-dependent, significant increase in BDNF concentration compared to control wells (Fig. 6A, B and Supporting Information Table 3). The amount of BDNF released from the (PDADMAC/HP)_3_/PDADMAC/BDNF multilayer (1120.4 ± 352.8 pg/mL and 2649.5 ± 213.2 pg/mL, respectively) was significantly higher than from corresponding (PDADMAC/HP)_4_/BDNF multilayer (31.13 ± 11.6 pg/mL and 1375.7 ± 350.6 pg/mL, respectively). To exclude the influence of SH-SY5Y on BDNF levels, we additionally included in the bulk analysis, without SH-SY5Y adhered. The observed effects were constant regardless of SH-SY5Y presence.

Additionally, an ELISA assay was carried out on supernatant samples collected directly after 15 min of adsorption of BDNF to verify the amount of residual BDNF (BDNF not adsorbed on the multilayers during cell culture plate preparation) (Supporting Information Fig. 3). The results indicate a vast amount of protein is not effectively adsorbed on the multilayers, especially the (PDADMAC/HP)_3_/PDADMAC and is therefore washed out.

### BDNF uptake in SH-SY5Y cells

**Figure 7:**
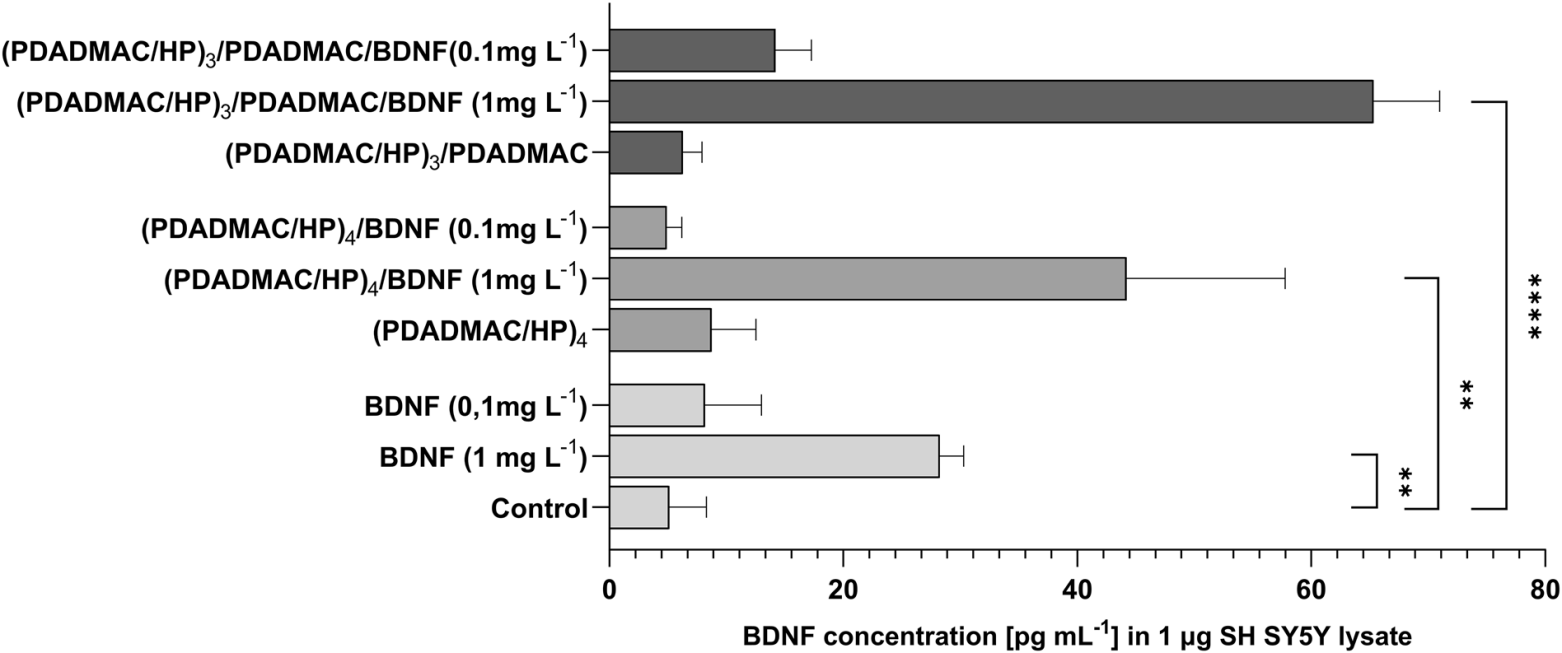
The effect of PDADMAC/HP and PDADMAC/HP/BDNF multilayers on BDNF uptake to SH-SY5Y cells. Previously, the SH-SY5Y cell line was exposed to various concentrations of BDNF (0.1 mg/L or 1 mg/L) and the (PDADMAC/HP)_3_/PDADMAC or the (PDADMAC/HP)_4_ multilayer for 24 h. As a control, cells were not exposed to BDNF and/or multilayers. After incubation, cell lysate was collected, and BDNF concentration was measured in 1 μg total cell lysate with the ELISA test. (**A**) Column graph showing mean ± SD of BDNF concentration (pg/mL) in 1 µg of cell lysate in each setting (n = 3). For a detailed table containing the statistical significance of tested variables, see Supporting Information Table 4. Statistical analysis was determined using Kruskal–Wallis, one-way ANOVA, and Two-Way ANOVA: ****p < 0.0001, ***p < 0.001, ** < 0.01, *p < 0.05.)

In order to test whether the cells are able to uptake BDNF released from the layers, after 24 h we have collected protein lysates of the cells incubated as described in previous sections. As ‘uptake’ we understand any amount of BDNF detected in cell culture lysates disregarding the exact mechanism by which the neurotrophin had entered the cell. The protein concentration was measured as described in the “Materials and methods” section. A total of 1 μg of cell protein lysate was analyzed. Generally, cells seemed to be able to uptake BDNF absorbed on the multilayers, at least we found more BDNF in cell lysates that were grown on multilayers with adsorbed BDNF compared to control (Fig. 7 and Supporting Information Table 4). The concentration-dependent amount of BDNF in lysates was significantly increased in both types of multilayers terminated with 1 mg/L BDNF, and also in cells that were treated with |1 mg/L BDNF only. Overall, the highest amount of BDNF was detected in cells that were growing on the (PDADMAC/HP)_3_/PDADMAC/BDNF (1 mg/L) multilayer (mean concentration 65.34 ± 5.64 pg/mL *vs* 28.25 ± 2.04 pg/mL in BDNF 1 mg/L and *vs* 44.22 ± 13.54 pg/mL in the (PDADMAC/HP)_4_. Our data suggest that the uptake of BDNF was more facilitated in the cells that were grown on the (PDADMAC/HP)_3_/PDADMAC/BDNF than it was for the cells grown on the (PDADMAC/HP)_4_ multilayer.

### Determination of lipid peroxidation

**Figure 8:**
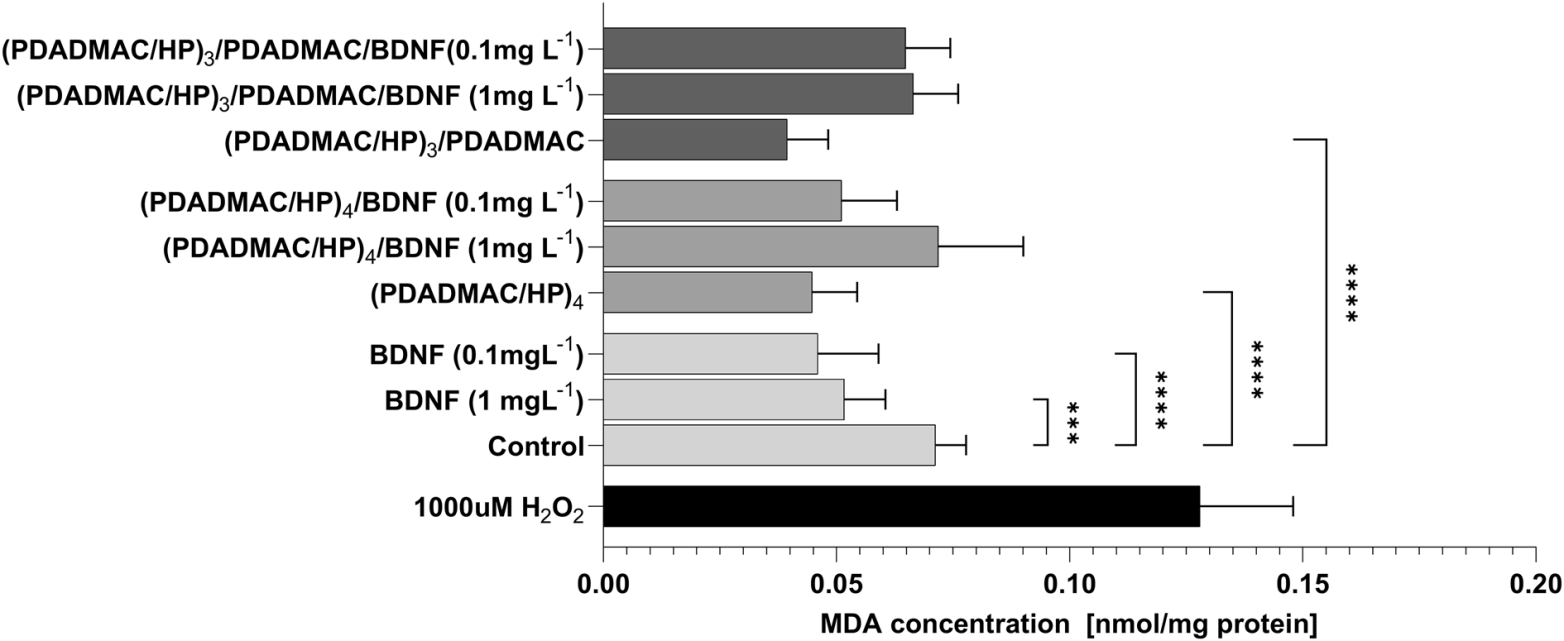
Assessment of MDA concentration in cell supernatants obtained after adsorption on BDNF, PDADMAC/HP, and PDADMAC/HP/BDNF. The SH-SY5Y cell line was exposed to various concentrations of BDNF (0.1 mg/L or 1 mg/L), (PDADMAC/HP)_3_/PDADMAC, (PDADMAC/HP)_3_/PDADMAC/BDNF (1 mg/L), (PDADMAC/HP)_3_/PDADMAC/BDNF(0.1 mg/L), (PDADMAC/HP)_4_, (PDADMAC/HP)_4_/BDNF (1 mg/L), (PDADMAC/HP)_4_/BDNF (0.1 mg/L) layers with adhered SH-SY5Y cells, for up to 24 h. MDA concentration was normalized to the total protein concentration of individual supernatant samples. Column graph showing mean ± SD of MDA concentration (nmol/mg protein) in each setting (n = 12). For a detailed table containing the statistical, significance of tested variables, see Supporting Information Table 5. Statistical analysis was determined using Kruskal–Wallis, one-way ANOVA, and Two-Way ANOVA: ***p < 0.001, ** < 0.01, *p < 0.05.

The concentration of MDA-(TBA)_2_ complex was examined to monitor how different polyelectrolyte (PDADMAC/HP) layers and the presence of BDNF (0.1 mg/L or 1 mg/L) influence oxidative stress by lipid peroxidation. The assay was calibrated from 0.05 to 0.2 µM TEP, and the results were normalized to the total protein concentration of individual supernatant samples (Fig. 8 and Supporting Information Table 5).

Using the reverse phase HPLC technique, we noticed a statistically significant reduction in MDA levels in (PDADMAC/HP)_4_ and (PDADMAC/HP)_3_/PDADMAC multilayers in comparison to the control. MDA concentration in control (0.071 + − 0.006 nmol/mg protein), was almost twice the value from the (PDADMAC/HP)_3_/PDADMAC. The decrease of MDA concentrations was also present in cell supernatants after BDNF treatment (0.1 mg/L and 1 mg/L) without layers (0.05 + − 0.01 nmol/ mg and 0.05 + − 0.001 nmol/mg, respectively). Additionally, (PDADMAC/HP)_4_/BDNF (0.1 mg/L) and.

(PDADMAC/HP)_4_/BDNF (1 mg/L) layers showed BDNF concentration-dependent difference (0.05 + − 0.01 nmol/mg and 0.07 + − 0.02 nmol/mg). In contrast, we have not observed such differences with the (PDADMAC/HP)_3_/PDADMAC (0.064 + − 0.001 nmol/mg and 0.066 + − 0.001 nmol/mg) and BDNF without layers. Cells treated with H_2_O_2_ were used as a positive control of response to oxidative stress.

### The analysis of cell morphology

The SH-SY5Y cell line is heterogeneous, consisting of both S-type (epithelial-like) and N-type (neuroblastic) undifferentiated cells. N-type cells express characteristic neurites, in contrast to S-type cells, which lack these features^49^. As the addition of BDNF in reduced or serum-free medium is a common method for differentiating SH-SY5Y cells into mature neurons, we aimed to assess their differentiation status. However, we did not observe any differences in cell characteristics after the addition of BDNF at any concentration. Due to the limited scope of this publication, we were unable to conduct molecular analysis to determine the differentiation state of the cells.

**Figure 9:**
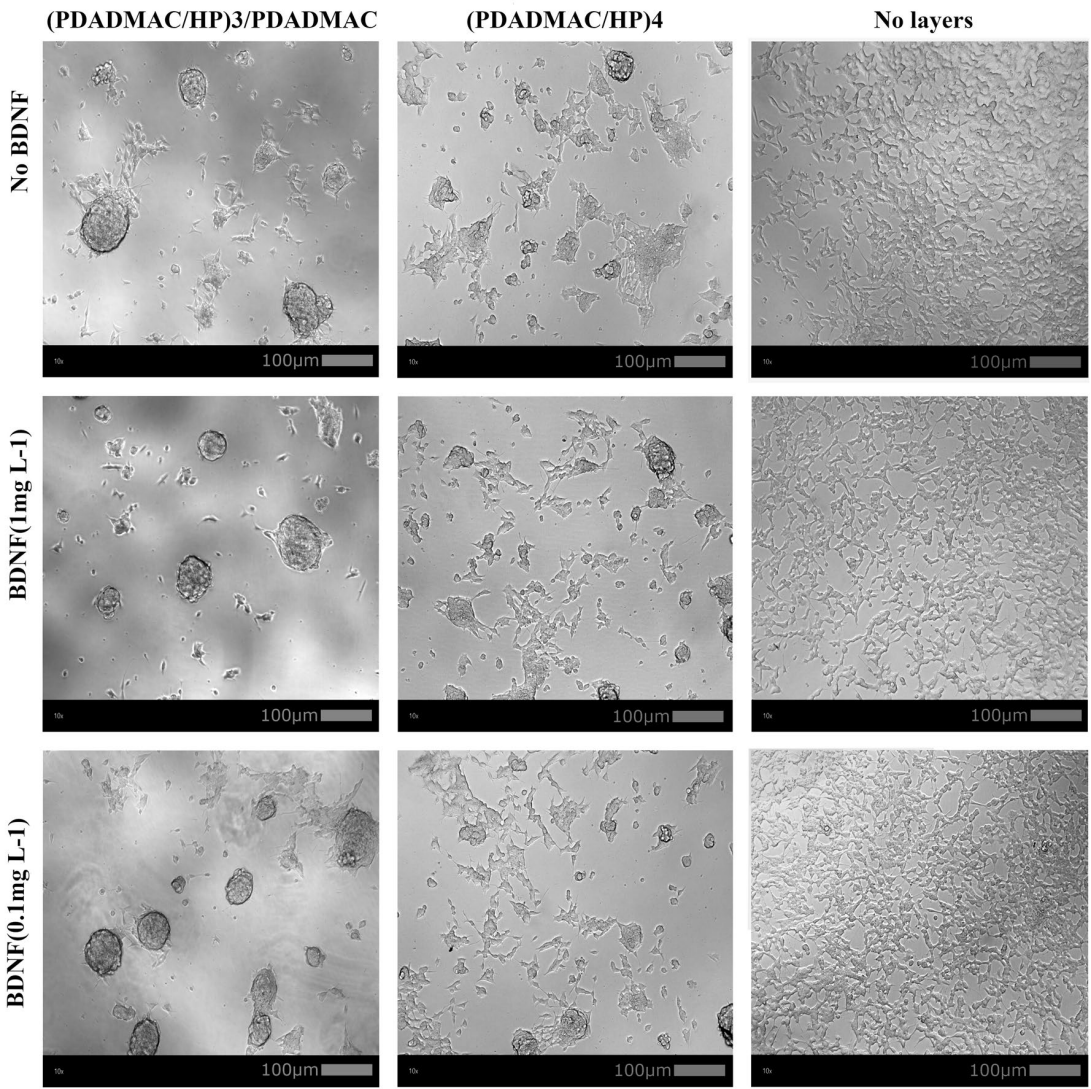
The effect of PDADMAC/HP and PDADMAC/HP/BDNF multilayers on SH-SY5Y cells’ morphology. As previously, SH-SY5Y cell line was exposed to various concentrations of BDNF (0.1 mg/L or 1 mg/L) and (PDADMAC/HP)_3_/PDADMAC, (PDADMAC/HP)_3_/PDADMAC/BDNF (1 mg/L), (PDADMAC/HP)_3_/PDADMAC/BDNF (0.1 mg/L), (PDADMAC/HP)_4_, (PDADMAC/HP)_4_/BDNF (1 mg/L), (PDADMAC/HP)_4_/BDNF (0.1 mg/L) layers and incubated for 8 days. As a control, cells were not exposed to BDNF and/or multilayers. After incubation, pictures of the cells were taken using a Progres Gryphax BETRIA camera (Jenoptic) from under the Leica DMIL LED microscope at 10×/0.25 PH1 and 20×/0.35 PH1(Leica) magnification.

We noted the presence of numerous spheroid-like cell structures in both the (PDADMAC/HP)_3_/PDADMAC and (PDADMAC/HP)_4_ multilayers (Fig. 9). These spheroid-like structures appeared to be more prominent and abundant in cells cultured on the (PDADMAC/HP)_3_/PDADMAC. Some of these characteristics were observable as early as day 4 post-seeding (see Supporting Information Fig. 4).

## Discussion

Many studies^50–55^ reported that biomaterials based on PEM require understanding the type of interactions between the polyelectrolyte films and cell surfaces. As discussed elsewhere, the adsorption of proteins and cells on biodegradable surfaces can significantly drive and modulate in vivo transport in biological fluids. Here, we characterize a nano-bio interface, which is a key challenge to determine whether biodegradable polymers bind to secreted cell products or to the cell membrane and produce a system to damage neuroblastoma cancer cells.

Initially, we characterized Lastly, the characteristics in situ by determining their physicochemical properties, such as the zeta potentials and the coverages of subsequent layers forming PEM and BDNF-coated PEM. Then, the effect of PEMs and PEM containing BDNF on the viability and morphology of neuroblastoma cells was evaluated.

As was shown in Fig. 1, periodic variations in the zeta potential between positive and negative values appear for the multilayers terminated by PDADMAC and HP, respectively. The sign of the zeta potential of the PDADMAC layers agrees with the sign of the zeta potential of PDADMAC in bulk, as measured by electrophoresis^56^. However, the zeta potential value of the PDADMAC layer adsorbed on silica was lower than the zeta potential of PDADMAC molecules determined in bulk for the same ionic strength (of 0.01 M) and pH (5.8). A similar observation was reported for the PDADMAC layer adsorbed on mica^55^. Interestingly, Zhou et al.^57^ evaluated the zeta potential of HP-terminated multilayers at pH 5.8 and found it to be the same. It is worth noting that the addition of BDNF changed the zeta potential values of both PDADMAC-terminated and HP-terminated films. The success of BDNF adsorption onto the polyelectrolyte films can be explained by electrostatic interaction between strongly positively charged PDADMAC, negatively charged HP, and slightly positively charged BDNF molecules as well as by the heterogeneous distribution of electrical charge over BDNF molecules^23^. These observations also agree with the results reported by Wasilewska and Adamczyk, who observed anomalous adsorption of negatively charged fibrinogen molecules on negatively charged mica at pH 7.4. This effect was explained in terms of heterogeneous charge distribution on fibrinogen molecules^58^.

Both PDADMAC-terminated and HP-terminated thin films remained stable throughout the experiments, indicating their suitability as effective carriers of BDNF molecules. Diffusion of the negative HP chains from the region of the film near the solution interface, through the outermost PDADMAC and BDNF layers, and then exposing the HP chains into the solution, can cause a slight decrease in the zeta potential of the PDADMAC-terminated layer supplemented by BDNF (see Fig. 1B). The diffusion of various molecular masses of poly (styrene sulfonate) chains within PDADMAC/poly(styrene sulfonate) films was thoroughly studied in Ref.^59^. The authors found that whole-molecule diffusion was faster in the region of the film near the solution interface. However, it should be noted that due to low positive charge, BDNF can also be adsorbed reversible on a positively charged PDADMAC layer.

Practically no desorption was noted when BDNF was adsorbed onto HP-terminated multilayers. However, it is not possible, without additional measurements, to unequivocally attribute the differences in layer stability against washing. OWLS measurements indicated the reversible adsorption of BDNF on PDADMAC-terminated films occurring under steady-flow conditions. It is worth emphasizing that even if PDADMAC alone did not work as an effective carrier for BDNF, PDADMAC, and HP can work as effective building blocks forming stable, homogenous films^39^. When these coatings are terminated by HP, the multilayers can also serve as an effective carrier for BDNF.

SPM results agree with the ELISA assay, where BDNF was adsorbed on PDADMAC-terminated and HP-terminated PEM under static conditions (no flow) (Fig. 1). One can postulate that the electrokinetic (uncompensated) charge on the BDNF molecules and the PBS composition have an impact on protein adsorption. The low density of charges on BDNF molecules as well as the presence of Na^+^, Cl^−^, K^+^, HPO_4_^2−^, and H_2_PO_4_^−^ in PBS solution, causes the formation of hydrogen bonds between BDNF molecules, encouraging aggregation or enhanced charge heterogeneities leading to localized short-range attraction and long-range electrostatic repulsions. BDNF, like most proteins, may easily self-associate into reversible oligomers of different sizes, depending on the association tendency, concentration, and other solution conditions described previously for various types of protein present in the literature^60,61^. The surface properties of PEM have been found to influence their cytotoxicity, which varies between cell types as well as polyelectrolyte functionalization with various terminal groups. The variations in cell response found in cytotoxicity studies can be explained by different physicochemical properties of PEM and their interactions with cell membranes, which remains poorly understood.

According to our previous work^11^, the BDNF molecule has a slightly positive electrokinetic charge under physiological conditions, which explains a significant increase of BDNF adsorption on the negatively charged HP layer. Decreasing the zeta potential of the PDADMAC-terminated multilayer (see Fig. 1A) and practically no changes in PEM coverage due to BDNF supplementation (see Fig. 3A) indicate that BDNF reversibly adsorbs on PEM under flow conditions.

Contrary to OWLS and SPM studies, in vitro measurements were made in static conditions. We determined residual BDNF concentration *versus* various PEM configurations using ELISA assay from samples collected after adsorbing BDNF. In no-flow experimental conditions, the adsorption of BDNF on the (PDADMAC/HP)_3_/PDADMAC was 85%, while the studies showed unequivocally that PEM significantly reduced the proliferation of neuroblastoma cells during 24 h of the experiment. (PDADMAC/HP)_3_/PDADMAC multilayers significantly decreased the neuroblastoma's cell viability by 28%. It agreed with the observation that the increased release of BDNF in the cell culture medium was also observed for the positive charge-terminated layer regardless of SH-SY5Y presence. This excludes the effect of BDNF production by the neuroblastoma cell line, which is in good accordance with the expression of BDNF in cancerous and normal tissues^62^.

In our study, we observed a decrease in cell viability, so we assume that there are internal or external stress factors facilitating apoptosis^63^. It is possible that apoptosis could be attributed to a malfunction in cellular protein turnover maintenance (proteostasis), considering an overrepresentation of a single protein type (BDNF) within cell^64,65^. Alternatively, the abundance of exogenous BDNF in the environment might trigger low-affinity BDNF receptor p75NTR and downstream pathways related to apoptosis, which is uncommon and unlike, but possible^1,12–14^. Nevertheless, the BDNF concentrations employed in this study are significantly lower than those employed in other research, yet we observe an effect on cell viability and morphology. One potential explanation is that PEMs may prolong the half-life of BDNF, making it more accessible and allowing the use of lower concentrations to exert a biological effect on cells. Our experimental results seem to support this notion, as when cells are grown with the addition of BDNF but without PEMs, the BDNF levels in both cell culture medium and cells are significantly lower after 24 h compared to using the same concentration adsorbed on the layers. Lastly, the characteristics of the multilayers themselves could potentially contribute to cell death. It was shown that PDADMAC-dominated surfaces in a serum-free medium were extremely toxic towards cells, as unbound PDADMAC surfaces may harm the cells in medium^66^. However, the cytotoxicity of PDADMAC seems to depend on the composition of PEM and also cell type used. Certain publications report a positive effect of PDADMAC-based polyelectrolyte multilayers, for instance, poly(styrene sulfonate) /PDADMAC^67,68^ were shown to improve cell attachment, viability, and spreading of fibroblasts. On the contrary, PDADMAC-terminated PEM (PDADMAC/poly(styrene sulfonate) was not a compatible surface for muscle cells^69^. Our data demonstrate that PDADMAC-terminated PEMs did not exhibit toxicity toward SH-SY5Y cells. Furthermore, the increased cell death may be a consequence of enhanced spheroid-like structure formation, as not all cells within the population may be capable of such formation.

To our knowledge, oxidative stress has not yet been studied in PEM composed of charged PDADMAC and HP, obtained by the layer-by-layer method. Liu et al.^70^ have studied the antioxidative potential of the film made by biodegradable polymers carboxymethylcellulose sodium (CMC) with chitosan (CS). The authors indicated that the PEM film’s free radical scavenging rate in 30 min was 78.62%. Sharma et al.^71^ used hydroxyl-terminated polyamidoamine (PAMAM) dendrimers and *N*-acetyl cysteine (NAC) with triphenyl-phosphonium (TPP) to synthesize dendrimer-based therapeutic able to exhibit a modest decrease in superoxide levels in vitro.

In this study, we found that cancer cells growing on (PDADMAC/HP)_4_ and (PDADMAC/HP)_3_/PDADMAC layers exert a statistically significant reduction in concentrations of products of lipid peroxidation in comparison to control. Moreover, we also observed a reduction of MDA level in cell supernatants after a BDNF treatment without layers compared to controls. It is known that decreasing levels of BDNF in human serum are strictly related to higher MDA concentration and the occurrence of neurodegenerative diseases^72,73^, which is in line with our observation. Additionally, Bouvier et al., noticed that BDNF increased Nrf2 translocation to the nuclear compartment, constantly activating antioxidant mechanisms. This leads to the conclusion that neurotrophins significantly impact oxidative stress. Our study reveals that both PEM HP-terminated and PDADMAC-terminated have similar antioxidant effects. Moreover, we confirm that the MDA level in SH-SY5Y decreases, while cells are treated solely with 0.1 and 1 mg/L BDNF.

SH-SY5Y cells growing in a monolayer are well described in the literature, as well as the methods for their differentiation^48^. It was shown by multiple authors that the removal of serum from the cell culture medium combined with BDNF treatment would induce the differentiation of N-type cells into neuron-like cells, which could be detected with microscopy^74^. In a similar study, Zhou et al., examined the effect of BDNF adsorbed on polyelectrolyte multilayer consisting of positively charged poly-l-lysine and negatively charged HP on poly-e-caprolactone film prepared by layer-by-layer method. Immobilized BDNF caused an increase in neurite length and cell number of neural progenitor cells^75,76^. However, we did not observe a mature neuronal phenotype in cells exposed to BDNF. Typically, studies of this nature in literature analyze molecular mechanisms and markers to provide more comprehensive insights.

Moreover, in the course of our experiments, we observed that starting from day 4 post seeding onto layers, SH-SY5Y cells began to form spheroid-like structures. This would suggest the promotion of self-assembly of the cells on the layers containing BDNF. This effect is particularly well pronounced in cells that were growing on the (PDADMAC/HP)_3_/PDADMAC/BDNF multilayer, suggesting that high amounts of BDNF in the environment and within the cell, supported by PDADMAC-terminated multilayer, enhance organoid formation by cells. Typically, in order to achieve three-dimensional cell structure in vitro, researchers use techniques discouraging cells’ free movement, such as culturing cells in a hanging drop, on a scaffold, or on a surface covered with hydrogel^77^. Similarly, the PDADMAC/HP-terminated multilayers might promote 3D structure formation. The initial attachment of a cell to the cell plate surface relies heavily on electrostatic interactions. Typically, the cell membrane carries a negative electric potential, which can range from − 10 to − 90 mV, depending on the cell type^78^. In our study, we demonstrated that the adsorption of BDNF onto PEMs terminated with PDADMAC increases the zeta potential from 14 to − 2 mV. This change in zeta potential may potentially discourage cellular attachment, similar to the conditions in low-attachment cell culture plates, thereby promoting the formation of spheroid-like structures. 3D cell culture is of great importance for the development of more precise neurodegenerative disease models. Therefore, the formation of spheroids caused by the layers could be especially interesting for future research as an in vitro model of neurodegeneration.

## Conclusions

PEMs, comprising cationic (PDADMAC) and anionic (HP) polyelectrolytes, were successfully produced for effective BDNF delivery. The application of BDNF-PEM coacervates was tested in the environment of human neuroblastoma cell line SH-SY5Y.

It was found that BDNF reversibly adsorbs onto PDADMAC/HP multilayers, and the molecules can be easily released by changing the environment properties such as buffer ionic strength and pH. By interrogating the result from the physicochemical characterization of the biocompatible BDNF-loaded PEM layers, we facilitated an interpretation of experimental data derived from the applied biological techniques. The key finding of our study was that the (PDADMAC/HP)_3_/PDADMAC and (PDADMAC/HP)_4_ films have various impacts on BDNF release profile, cell viability, BDNF uptake, mitochondrial membrane potential, cell phenotype, and induction of lipid peroxidation.

It was proved that the biocompatible multilayers could be effectively produced by sequential adsorption of strongly positively and negatively charged polyelectrolytes from low polyelectrolyte concentrations (5 mg/L). Accordingly, besides the basic examinations, our results could also have practical applications. They can be successfully applied to form biocompatible multilayers (capsules, dressings) to further medical applications such as selective anticancer drug delivery or assessing the possibility of supportive PEM therapy for cancer developing in nerve tissues or neurodegeneration. We detected increased amounts of the protein of interest (BDNF) inside the cells grown on the (PDADMAC/HP)_3_/PDADMAC multilayer. This suggests that our system supports the protein's stability and allows cellular uptake. At the same time, both HP-terminated and PDADMAC-terminated multilayers can enhance 3D cell structure formation (spheroid-like), which suggests that by limiting cell-free movement, similar layers could be useful for future research as an in vitro model of neurodegeneration.

### Supplementary Information

Supplementary Information.

## Data Availability

The data supporting this study's findings are available from the corresponding authors upon reasonable request.
