# Supplementary material for: BDNF-loaded PDADMAC-heparin multilayers: a novel approach for neuroblastoma cell study
Source: Sci Rep. 2023 Oct 20;13:17939. doi: 10.1038/s41598-023-45045-y (PMC10589271; doi:10.1038/s41598-023-45045-y)

**SUPPORTING INFORMATION**

**BDNF-loaded PDADMAC-Heparin Multilayers: A Novel Approach for Neuroblastoma Cell Study.**

Maria Dąbkowska^1*^, Iga Stukan^2^, Bogusław Kowalski^1^, Wiktoria Donerowicz^1^, Alicja Szatanik^1^, Monika Wasilewska^3^, Małgorzata Stańczyk-Dunaj ^4^, Aneta Michna^3^

^1^ Independent Laboratory of Pharmacokinetic and Clinical Pharmacy, Pomeranian Medical University, Rybacka 1, 70-204 Szczecin, Poland

^2^ Department of General Pathology, Pomeranian Medical University, Rybacka 1, 70-204 Szczecin, Poland

^3^ Jerzy Haber Institute of Catalysis and Surface Chemistry, Polish Academy of Science, Niezapominajek 8,
 PL- 30239 Krakow, Poland

^4^ Department of Medical Chemistry, Pomeranian Medical University, Rybacka 1, 70-204 Szczecin, Poland

^*^ Corresponding author: [maria.dabkowska@pum.edu.pl](mailto:maria.dabkowska@pum.edu.pl)

**The thicknesses of macroion multilayers determined by OWLS.**

**
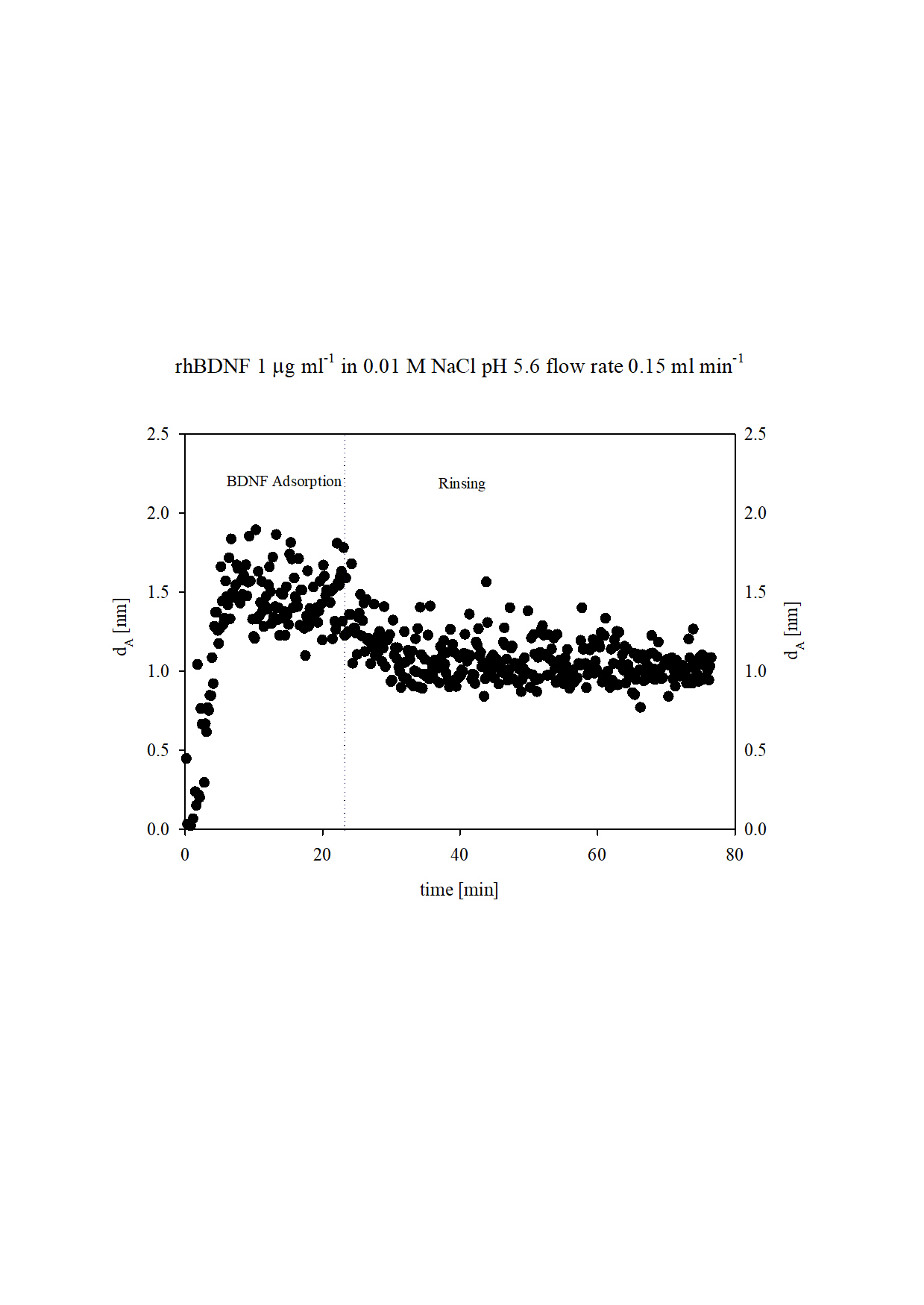
**

**Fig. 1.** The thickness of BDNF layer on silica sensor. Adsorption conditions: bulk concerntration of BDNF, cBDNF, was 1 mg L^-1^, I=0.01 M NaCl, pH 5.6


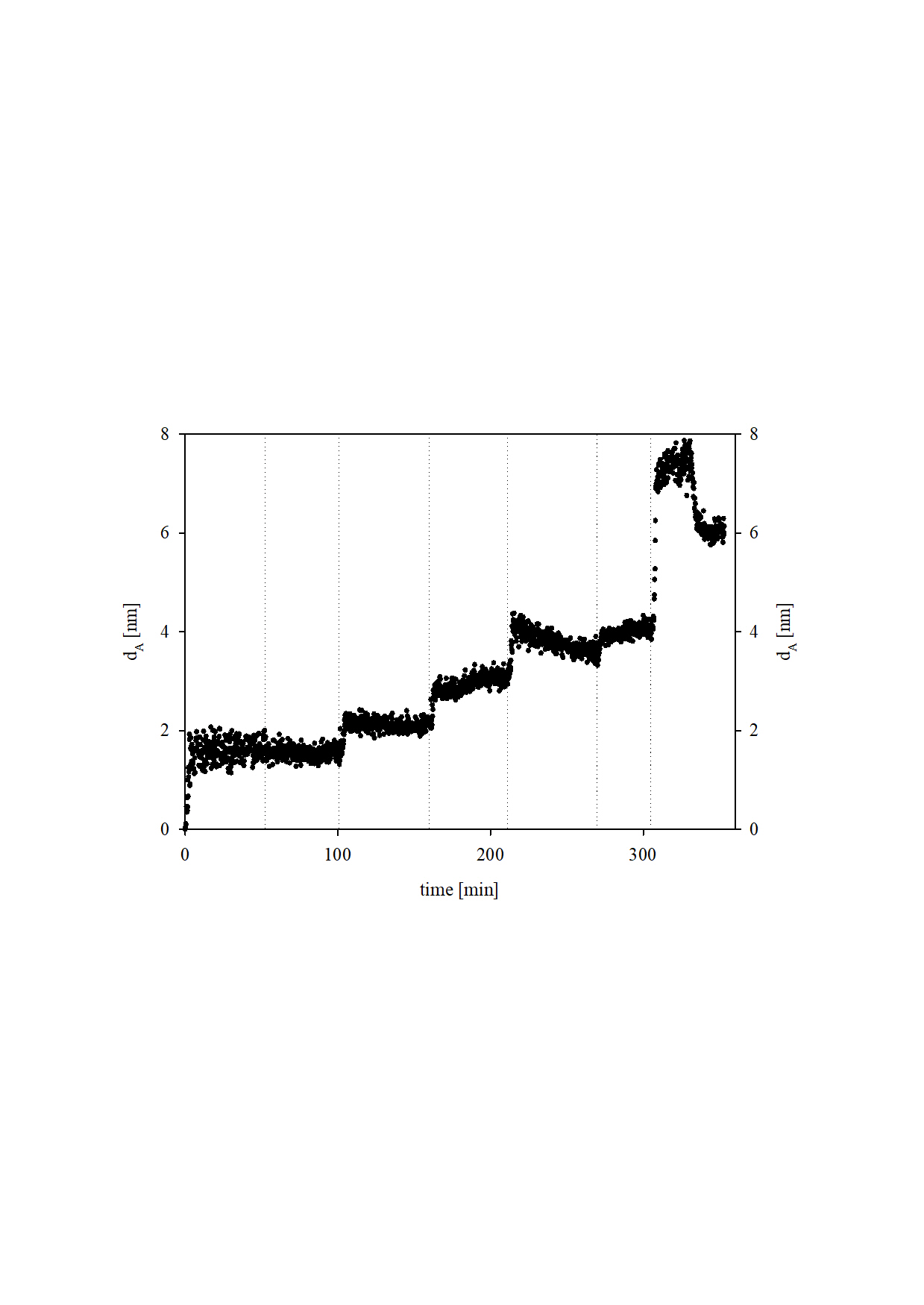


**Fig. 2.** The thickness of (PDADMAC/HP)3BDNF film on silica OWLS sensor. Adsorption conditions: bulk concentration of PDADMAC (HP), cPDADMAC = cHP = 5mg L^-1^; cBDNF = 1 mg L^-1^, I=0.01 M NaCl, pH 5.6

**
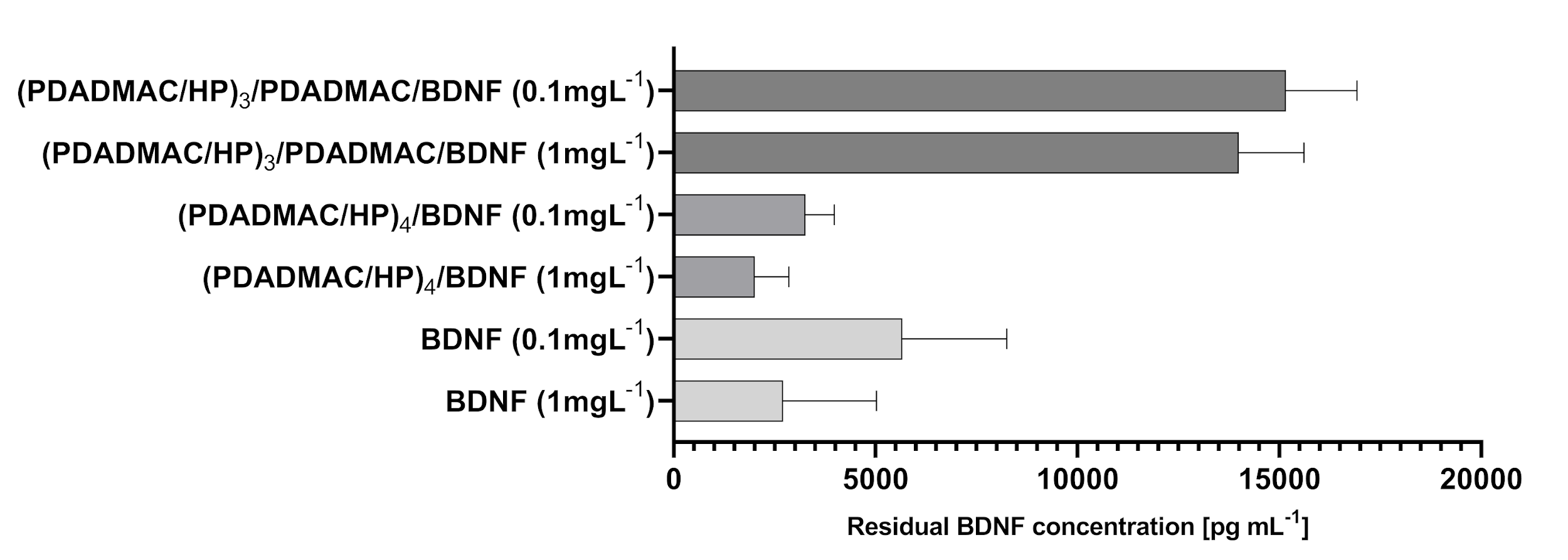
**

**Fig**. 3 **Residual BDNF measured with an ELISA assay from samples collected after adsorbing BDNF for 15 min. on various measurement configurations BDNF (0.1 mg L^-1^ or 1 mg L^-1^), (PDADMAC/HP)_3_/PDADMAC, (PDADMAC/HP)_3_/PDADMAC/BDNF(1 mg L-^1^), (PDADMAC/HP)_3_/PDADMAC/BDNF (0.1 mg L^-1^), (PDADMAC/HP)_4_, (PDADMAC/HP)_4_/BDNF
(1 mg L^-1^), (PDADMAC/HP)_4_/BDNF(0.1 mg L^-1^) layers.**

An ELISA assay was performed on supernatant samples collected after adsorbing BDNF for 900s on a previously prepared PDADMAC/HP multilayer. The samples include configurations of BDNF (0.1 mg L^-1^ or 1 mg L^-1^), (PDADMAC/HP)_3_/PDADMAC, (PDADMAC/HP)_3_/PDADMAC/BDNF (1 mg L^-1^), (PDADMAC/HP)_3_/PDADMAC/BDNF (0.1 mg L^-1^), (PDADMAC/HP)_4_, (PDADMAC/HP)_4_/BDNF(1 mg L^-1^), (PDADMAC/HP)_4_/BDNF(0.1 mg L^-1^) layers before adhering the SH-SY5Y cells were collected. The column graph shows the mean ± SD of BDNF concentration.

**
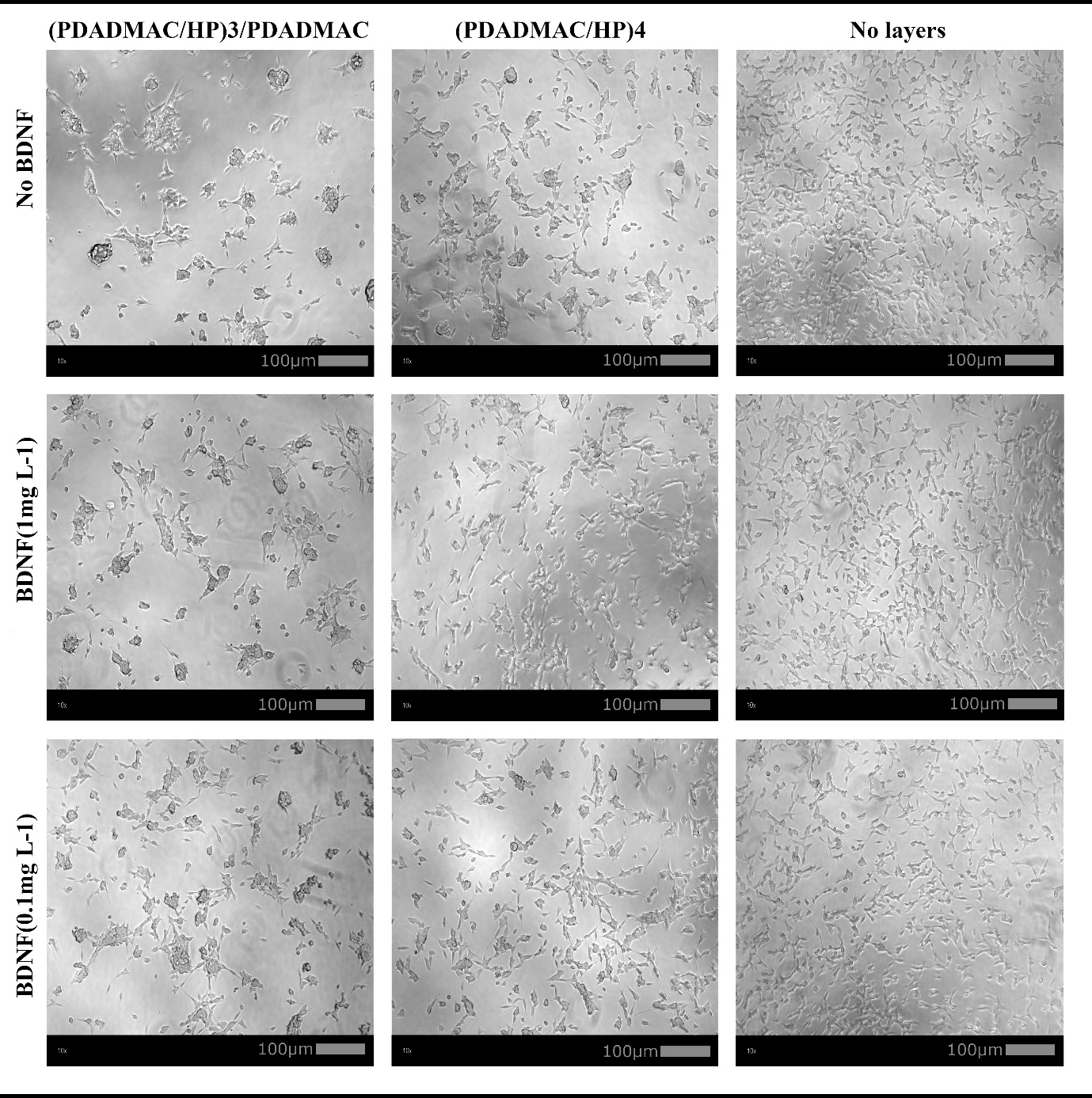
**

**Fig. 4.** **The morphology of SH-SY5Y neuroblastoma cell line after 4 days of incubation on multilayers with or without BDNF.** SH-SY5Y were seeded and incubated for 4 days on either (PDADMAC/HP)_3_/PDADMAC, (PDADMAC/HP)_3_/PDADMAC/BDNF (1 mg L^-1^), (PDADMAC/HP)_3_/PDADMAC/BDNF (0.1 mg L^-1^), (PDADMAC/HP)_4_, (PDADMAC/HP)_4_/BDNF (1 mg L^-1^), (PDADMAC/HP)_4_/BDNF(0.1 mg L^-1^) layers, no layers with the addition of BDNF (0.1 mg L^-1^ or 1 mg L^-1^) or no BDNF and no layers (control). Pictures of the cells were taken using a Progres Gryphax BETRIA camera (Jenoptic) from under the Leica DMIL LED microscope at 10x/0.25 PH1 and 20x/0.35 PH1(Leica) magnification

**Table. 1. The detailed statistical analysis of the effect of BDNF, PDADMAC/HP and PDADMAC/HP/BDNF multilayers on cell viability. Statistical analysis was determined using Kruskal-Wallis, one-way ANOVA and Two-Way ANOVA: ****p<0,0001, ***p< 0.001, **<0.01, *p<0.05.**


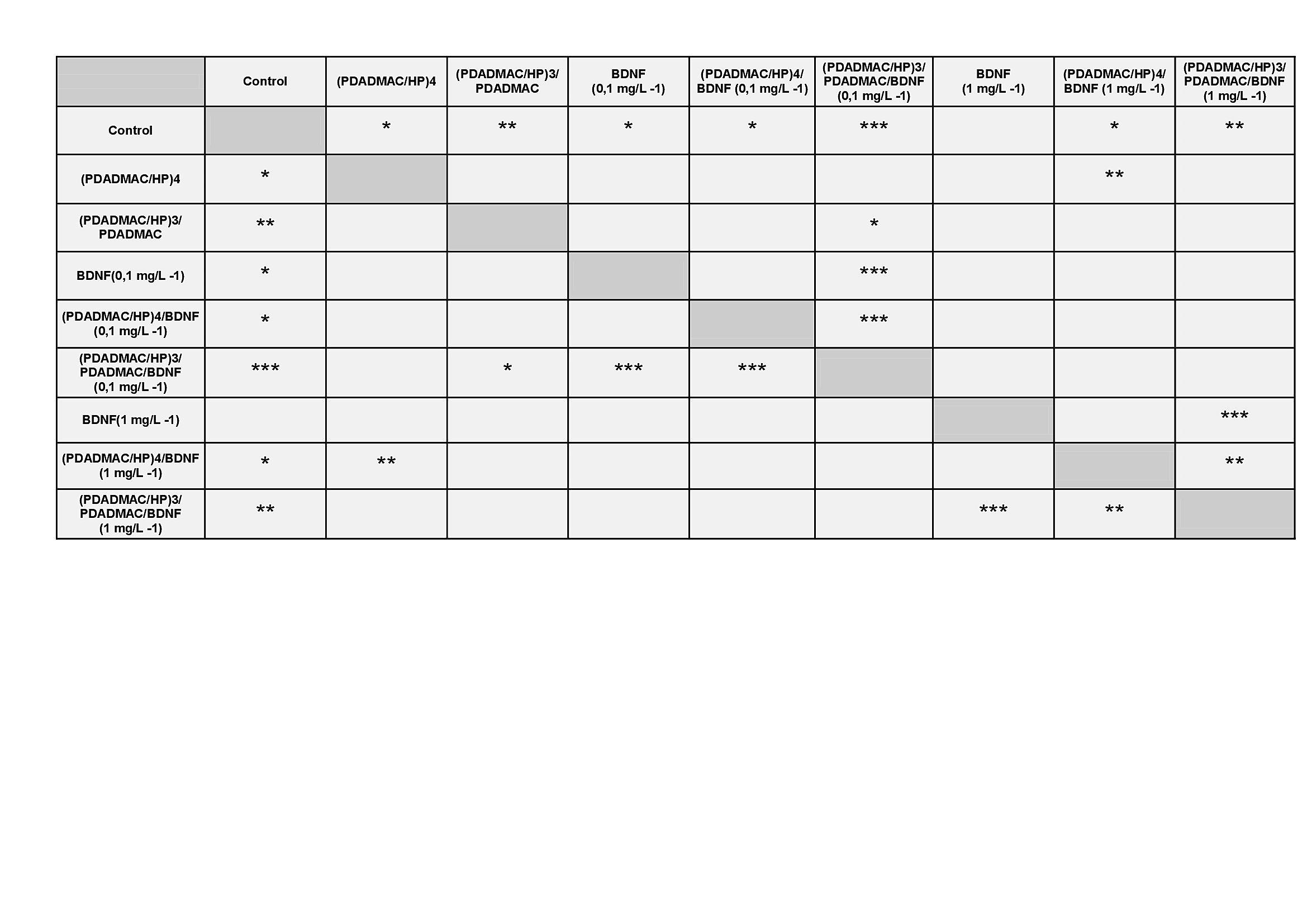


**Table. 2.** The detailed statistical analysis of the e**ffect of BDNF, PDADMAC/HP, and PDADMAC/HP/BDNF multilayers on mitochondrial membrane potential. Statistical analysis was determined using Kruskal-Wallis, one-way ANOVA and Two-Way ANOVA: ****p<0,0001, ***p< 0.001, **<0.01, *p<0.05.**


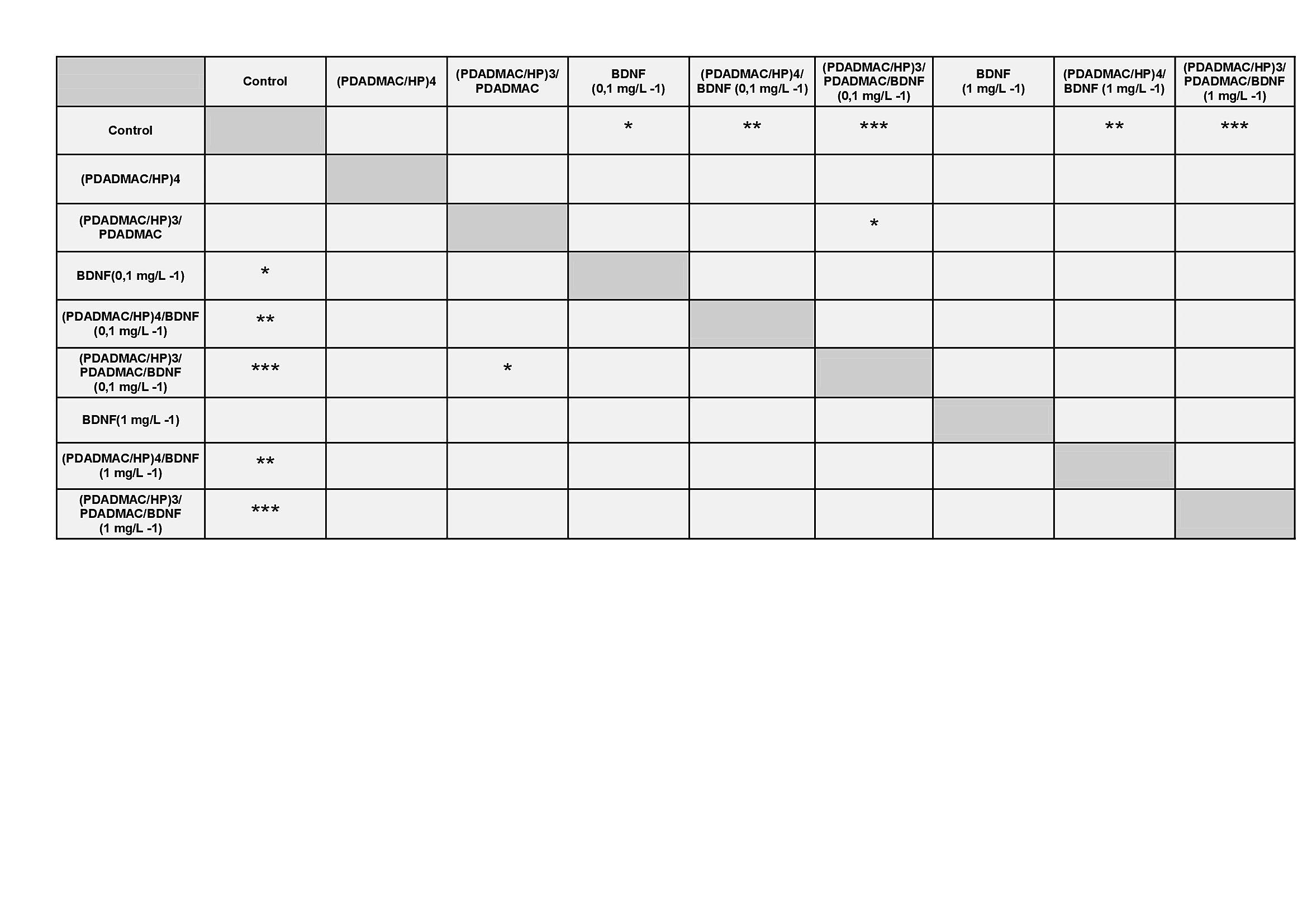


**Table. 3. The detailed statistical analysis of the effect of BDNF, PDADMAC/HP and PDADMAC/HP/BDNF multilayers on BDNF release to medium.Statistical analysis was determined using Kruskal-Wallis, one-way ANOVA and Two-Way ANOVA: ****p<0,0001, ***p< 0.001, **<0.01, *p<0.05**


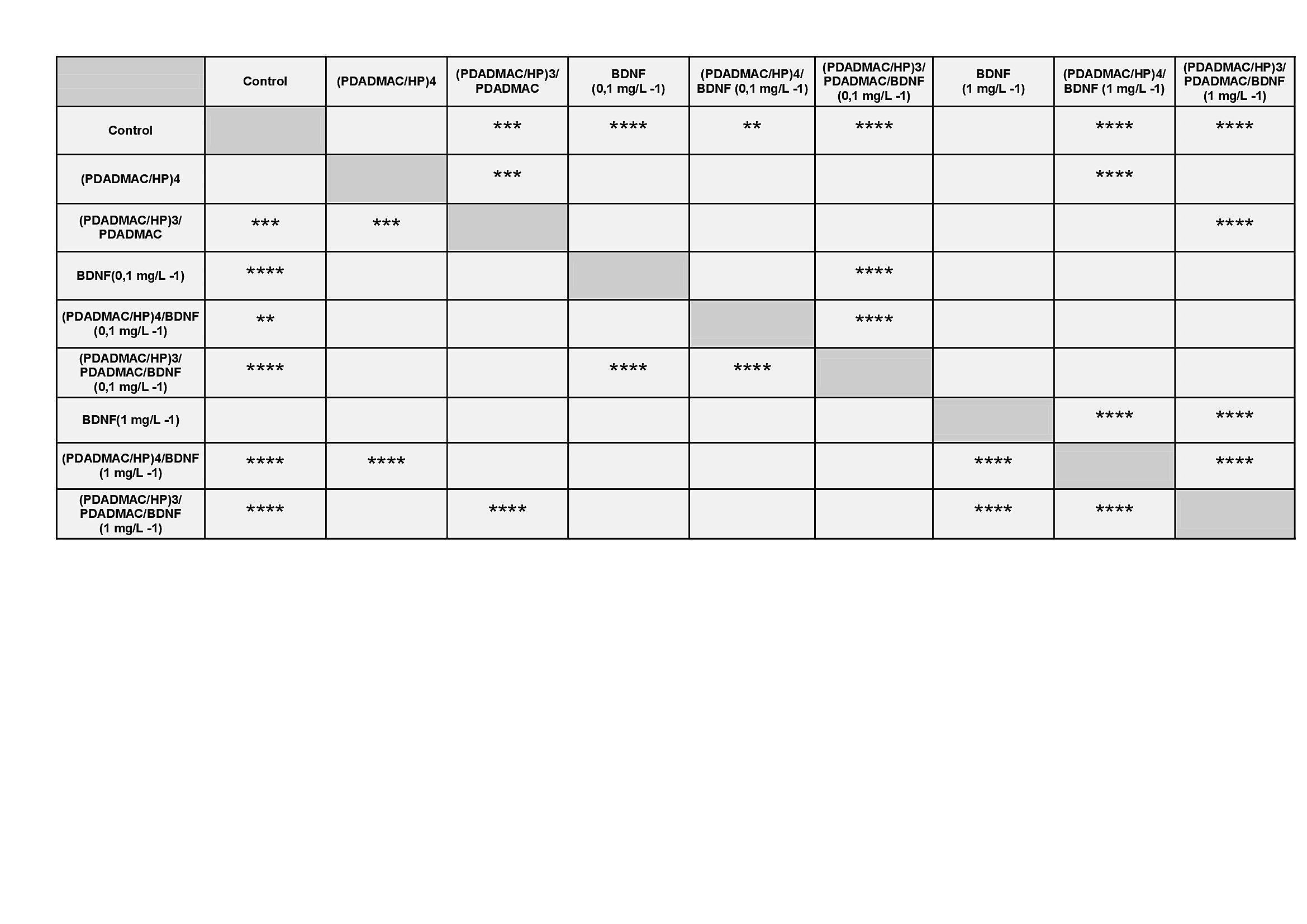


**Table. 4.** **The detailed statistical analysis of the effect of PDADMAC/HP and PDADMAC/HP/BDNF multilayers on BDNF uptake to SH-SY5Y cells.** **Statistical analysis was determined using Kruskal-Wallis, one-way ANOVA, and Two-Way ANOVA: ****p<0,0001, ***p< 0.001, **<0.01, *p<0.05.)**


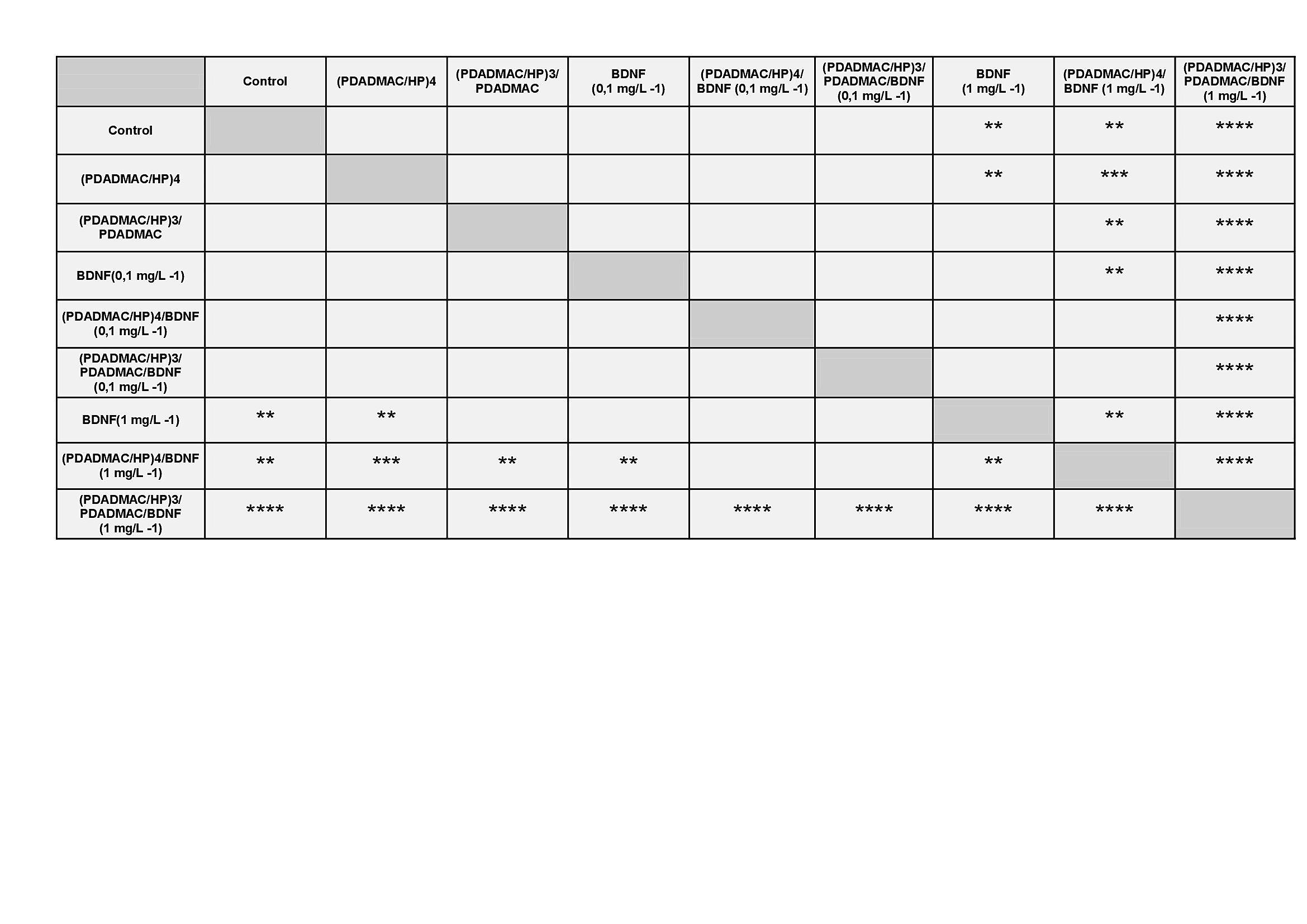


**Table. 5. Assessment of MDA concentration in cell supernatants obtained after adsorption on BDNF, PDADMAC/HP, and PDADMAC/HP/BDNF.** Statistical analysis was determined using Kruskal-Wallis, one-way ANOVA and Two-Way ANOVA: ***p< 0.001, **<0.01, *p<0.05.


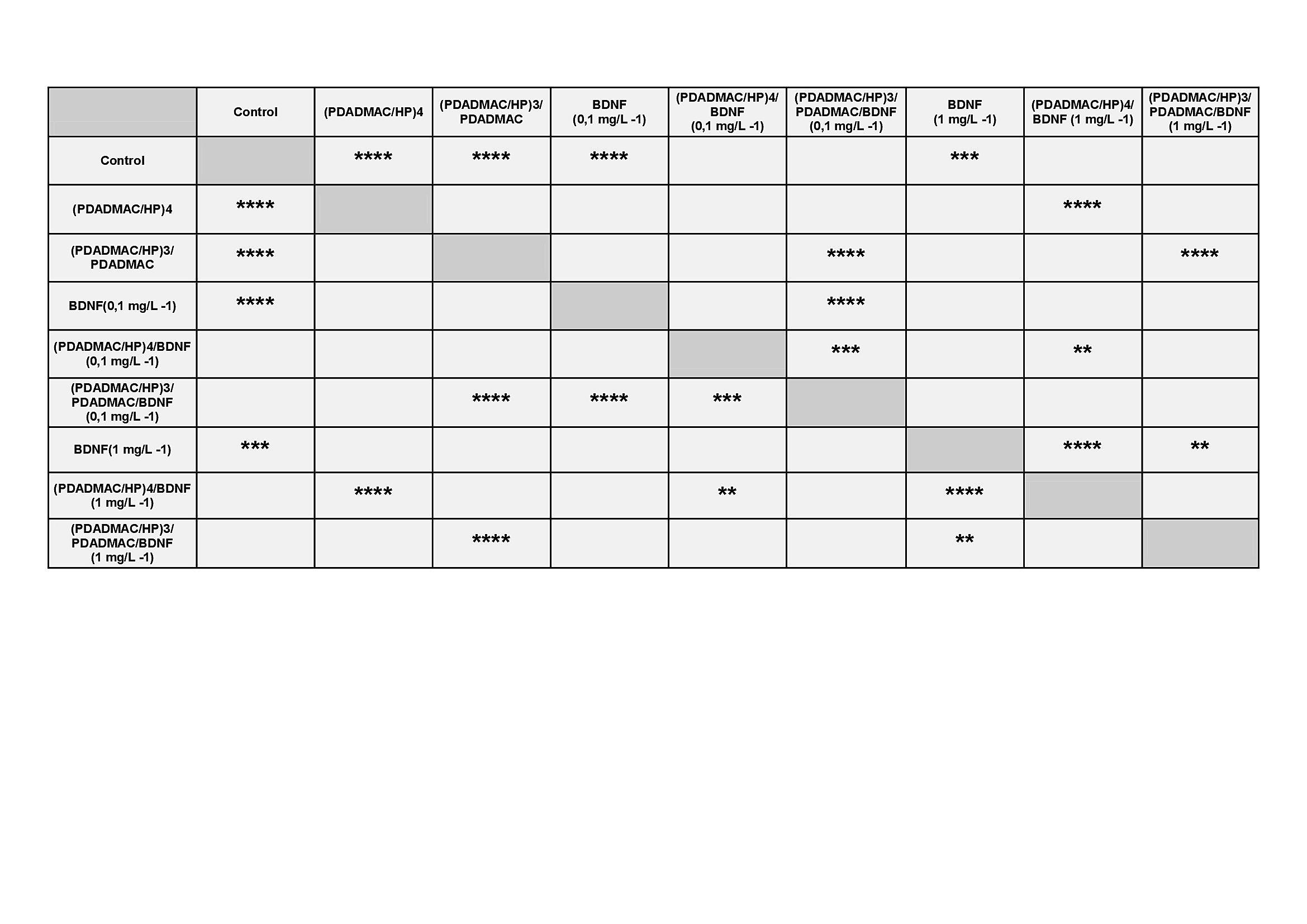

Supplement: Supplementary file 1 — Supplementary Information. [file 41598_2023_45045_MOESM1_ESM.docx]
